# Legacy tree-ring dataset reveals rapid use of panels in 17th-century Dutch and Flemish painting workshops

**DOI:** 10.1038/s40494-026-02737-8

**Published:** 2026-06-24

**Authors:** Kirsten Weterings, Marta Domínguez-Delmás, Sven Dupré

**Affiliations:** 1 Department of History and Art History, Utrecht University, Utrecht, The Netherlands; 2 Department of Archaeology, Cultural Heritage Agency of the Netherlands, Amersfoort, The Netherlands; 3 Functional Traits Group, Naturalis Biodiversity Center, Leiden, The Netherlands; 4 Faculty of Humanities, University of Amsterdam, Amsterdam, Netherlands

## Abstract

Dendrochronological research of panel paintings provides essential evidence for their dating and attribution. However, the uncertain interval between the tree’s felling and a painting’s completion, accounting for the transport, seasoning, and storage of the wood, hinders the accurate estimation of production dates. This study combines historical research with an analysis of dendrochronological data from over 1900 17th-century Dutch and Flemish panel paintings to examine the interval between tree felling and painting production, considering additional storage time at artist’s workshops. Results show that panels were typically used within 4.6 to 7.5 years of felling (0.3–5.6 years for French/Belgian/German, 1.0–6.1 years for Polish, and 6.1–9.6 years for Eastern Baltic oak). Paintings with boards from the same tree were completed within 1 to 1.8 years from each other. These findings serve to refine estimated production periods based on provenance of the wood and exact dendrochronological dates, aiding in the attribution of disputed artworks.

## Introduction

Determining the exact production time of paintings can be a decisive step in establishing key information about artworks, such as their attribution. Before the use of canvas became more widespread after the mid-17^th^ century, wooden panels were the preferred support in Northern European painting since the late Middle Ages. Oak (*Quercus* sp.) was the preferred species for panel paintings in the Low Countries, with few exceptions^1,2^. Because oak is suitable for dendrochronological dating, dendrochronological research can provide insights into the production period of Dutch and Flemish panels. Since the 1980s, there has been a constantly-growing body of dendrochronological case studies on seventeenth-century panels, and recent studies have contributed important new reference chronologies to determine the date and provenance of the timber used in these paintings^3,4^.

Dendrochronology is a dating method that correlates each tree ring in wood with the exact calendar year it was formed. When sapwood (i.e. the outermost part of the stem, just beneath the bark) is preserved in a panel, it enables dendrochronologists to estimate the felling date of the tree within a range of years. Oak trees have a predictable number of sapwood rings that decreases from northwestern to eastern Europe, making it possible to approximate the number of rings missing until the bark^5,6^. When sapwood is absent, dendrochronological research can only provide a *terminus post quem*, i.e., the earliest possible date *after* which the tree was cut. During the panel-making process, sapwood was deliberately removed from panels because, when dry, it is prone to degradation and woodworm infestation. As a result, art historians frequently still rely on signatures, stylistic features, archival records and other documentary evidence to determine a painting’s exact production date.

Understanding how much time elapsed between the felling of a tree and the completion of a painting is crucial to reliably estimate a painting’s production date. After the tree’s felling, some additional years must be accounted for the transport and drying of the wood. Up to the mid-17th century, most of the oak used in Dutch and Flemish ateliers originated from the Baltic region^3,4,7^. The wood arrived by ship to Dutch harbors as wainscots^8^, a timber product resulting from splitting oak stems several times along the grain^9^. Panels for paintings were produced by specialized woodworkers known as panel makers^10^, who sourced wainscots from timber markets and split or sawed them into thinner boards. The boards were then assembled into panels by selecting boards that matched the required dimensions (either custom sizes requested by the artists, or standard formats^11^), while minimizing material loss, to be used by a painter as a painting support.

**Fig. 1:**
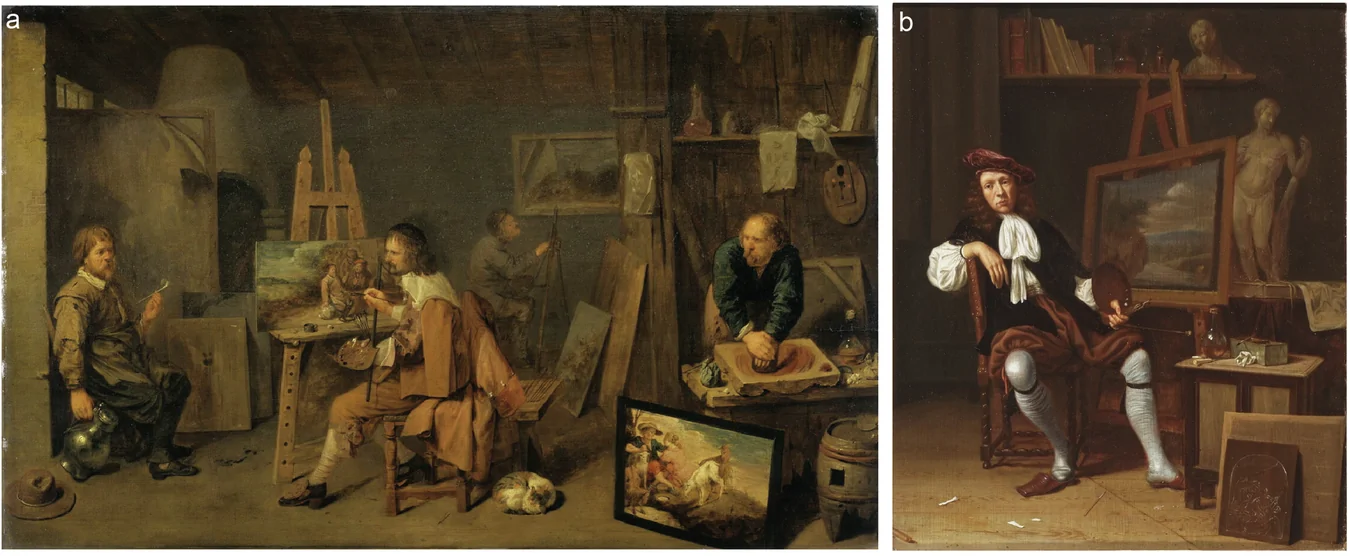
Seventeenth-century studio interiors depicting panels in various stages of preparation. **a** David Ryckaert (III), *Painter’s workshop with color grinder and posing model*, 1638, oil on panel, 59 ×95 cm, Louvre Museum (Paris), photo: Wikimedia Commons. **b** Michiel van Musscher, *Portrait of an Artist in his Studio*, 1665-1705. Oil on panel, 29 ×34.5 cm, Rijksdienst Cultureel Erfgoed, photo: Wikimedia Commons.

In addition to drying and transportation, historical sources suggest that painters kept a supply of prepared panels available for future use. Visual depictions of 17th-century studio interiors—such as David Ryckaert’s *Painter’s workshop with color grinder and posing model* and Michiel van Musscher’s *Portrait of an Artist in his Studio* (Fig. 1)—show unpainted wooden panels among the materials present in artists’ workshops^12^. Documentary evidence from artists’ inventories suggests larger quantities may have been stored elsewhere in the household, such as attics or storage rooms (Supplementary materials_Data). For example, the 1656 estate inventory of Jacob Jansz. van Velsen lists as many as “19 round, unpainted panels” and “30 unpainted oblong and square panels, both large and small” stored in the back attic^13^. Therefore, the possibility that panels were stored in the artist’s studio for a while must be considered when assessing the production time of paintings.

An early study from the 1970s estimated that the interval for the drying and transport of the wood was 8 ± 5 years in the 17^th^-century Low Countries, based on comparisons between the estimated felling date of the tree and the production date of a limited number of signed works: eight paintings retaining partial sapwood, and fourteen without sapwood^14^. Later publications report a shorter interval of 2–5 years during the 16^th^- and 17^th^-century period^1,2,15^, though these estimates are not accompanied by information about the datasets, sample sizes, or statistical analyses used to derive them. Furthermore, as the estimation of this interval was not the primary focus of these studies, they did not systematically consider factors like variation related to the provenance of the timber, or that painters may have stored panels for some time at their studios.

Here, we revisit the previously proposed intervals between the felling of trees and the completion of paintings, and investigate the consumption rate of panels at artist’s workshops. For this, we draw on a large-scale legacy dataset comprising thousands of dendrochronological tree-ring series from over 1900 panel paintings attributed to 16^th^- and 17^th^-century Dutch and Flemish artists. The primary focus is on the 17^th^ century, which represents a key transition phase in panel usage in the Low Countries, marked by shifting timber supply networks, a diversification from predominantly Baltic sources, increasing standardization of panel supports, and changing demands of the open art market. This period is particularly well suited for this analysis, as it comprises a substantial corpus of well-documented signed and dated works, providing a robust basis for statistical evaluation.

The legacy dataset used in this research derives from a large-scale archive assembled by Peter Klein (University of Hamburg) over the course of four decades, and represents one of the most extensive bodies of dendrochronological research on panel paintings currently available. Filtering the ones signed and dated by the artists and those for which the production date is known through historical documentation, our aims were:

i. to revise the estimated time elapsed between the cutting of the tree and the production of the painting, using boards that retain sapwood, and comparing them with the known production dates. This will provide empirical evidence to make accurate estimations about the *earliest possible* production time of panels (when sapwood is not present in the boards), or the *most likely* production time (when sapwood is present in some of the boards);
ii. to assess for how long painters may have stored panels. For this, we compare the production dates of signed and dated paintings made from boards from the same tree.

By integrating dendrochronological evidence with art-historical research, this study provides an improved understanding of the transport, seasoning time and consumption rate of oak wood for panel painting in the 17th-century Low Countries, thereby improving the current toolbox for the dating of artworks.

## Results

### Boards with sapwood

Of the 4101 boards in the legacy dataset that matched our selection criteria (see Dataset collection), a total of 501 boards were recorded to contain sapwood. From these, 93 derive from paintings signed and dated by the artists. The number of sapwood rings ranged from 1 to 23. Most of the boards (*N* = 61, 65.6%) were reported by Klein as being of Polish/Baltic origin, whereas the rest (34.4%) was reported as being of German/Netherlandish origin. Internal crossdating suggests that only one pair of boards with sapwood derives from the same tree. They were considered as one entry for further provenance analysis and the felling date estimates.

### Provenance analysis

**Fig. 2:**
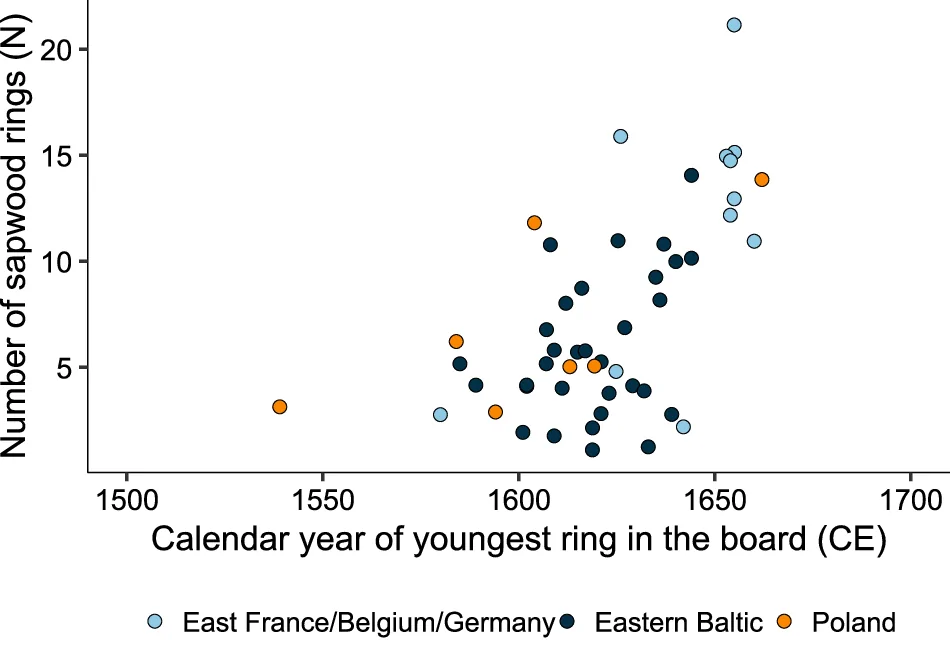
Number of sapwood rings on the boards and calendar date of the youngest sapwood ring present, indicated by region.

Determining the provenance of the wood is the first step to decide what sapwood statistics to use, as the number of sapwood rings in oaks has been observed to decrease from west to east in northern Europe^5^. Therefore, the provenance of the boards with sapwood was refined using new reference chronologies from the Baltic region^3^, the northeast of France, Belgium, and western Germany (Meuse, Moselle and Rhine River catchments)^16–18^. This analysis provided a more nuanced indication of the source area where the trees grew: the majority (*N* = 32, 62.7%) derive from the Eastern Baltic, a smaller group (*N* = 12, 23.5%) from Eastern France, Belgium and Western Germany, and a few from Poland (*N* = 7, 13.7%). The provenance of 36 boards could not be verified nor refined because the statistical results with the chronologies were inconclusive (*T*_BP_ < 5.0) (see Supplementary materials_Data). Based on those provenances, the sapwood statistics to be used are 6 to 18 rings for the Eastern Baltic (95% confidence interval)^6^, 8 to 38 for Eastern France, Belgium and Western Germany (95% confidence interval)^16^, and 9 to 24 for Poland (90% confidence interval)^19^. Plotting the number of sapwood rings present in each board against the calendar date of the youngest ring in the board reveals a slight upward trend in the amount of sapwood remaining in the boards over the course of the 17^th^ century (Fig. 2). In addition, there is a change in the prevalence of the Eastern Baltic provenance before and after 1650. After this date, the eastern French/Belgian/German provenance becomes dominant.

### Interval between tree felling and painting completion

**Fig. 3:**
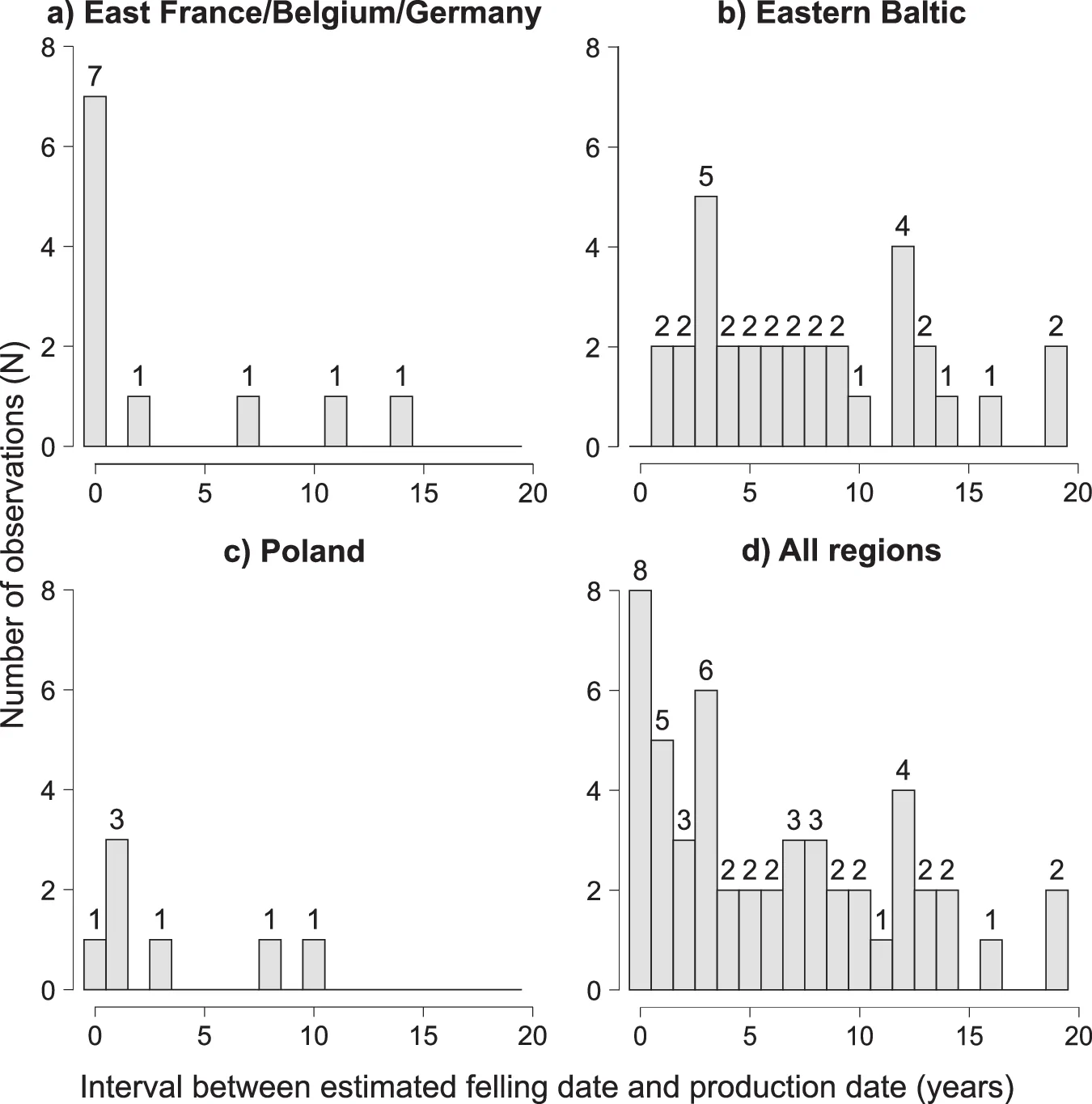
Interval between the estimated cutting date and production date. Frequency histograms of the interval between the mean estimated felling date and the production date by provenance (**a**–**c**) and all samples (**d**). The y-axis denotes the number of observations (N).

Having established the estimated felling date interval for every board with sapwood based on its provenance, the time period between the felling of the tree and the production of the painting can be approximated by subtracting the median of the felling date interval from the signed date of the painting. After removing outliers ( > 20 years difference between the median of the felling date interval and the production date, above the 99^th^ percentile of the collective data, see Discussion), the average time elapsed between the felling of the tree and the production of the painting can be estimated to be between 4.6 and 7.5 years within 95% confidence limits for the full dataset (*N* = 51) (Table 1). However, when considering the provenance of the wood, some regional differences emerge, as the span becomes 0.3 to 5.6 years for boards of central Western European origin, 1.0 to 6.1 years for boards of Polish provenance and 6.1 to 9.6 years for boards of Eastern Baltic provenance (Table 1, Fig. 3).

**Table 1:** Years spanned between the median estimated felling date of the trees and the production date of paintings

	Interval between mean estimated felling date and production date			
	*France/Belgium/Germany*	*Poland*	*Eastern Baltic*	*All*
N boards	12	7	32	51
Median span of years	0	1	7	5
Mean span of years	2.8	3.4	7.8	6.1
95% Confidence Interval (CI)	0.3–5.6	1.0–6.1	6.1–9.6	4.6–7.5
Std. Deviation	5.0	5.1	4.0	5.4
Absolute range of years	0 – 14	0–10	1 – 19	0–19

Bracket value in mean value represents the relative 95% confidence interval.

### Statistical identification of boards from the same tree

From the internal crossdating of the 4101 individual boards in the dataset, a total of 404 inter-board comparisons met the threshold of having a Student’s *t-*value (according to Baillie and Pilcher^20^; T_BP_) higher than 10, indicating they may derive from the same tree. In 184 (46%) of series-to-series comparisons, these statistical matches occurred between boards used for the construction of the same panel, while in the remaining 220 (54%) pairs of tree-ring series, the matching boards are found in different painting supports (Table 2). While many of the statistical matches occur in pairs (N = 174), there were at least 40 groups of three or more boards where each inter-board comparison meets the threshold.

**Table 2:** Overview of the internal crossdating results, listing counts of board-board comparisons with a Baillie-Pilcher t-value (T_BP_) > 10

Number of pairwise comparisons with T_BP_ > 10.0	
*Relationship between boards*	
Within the same panel (intra-artwork)	184
Within different artworks (inter-artwork)	220
*Artwork attribution*^a^	
Attributed to the same artist (or workshop)	111
Attributed to different artists (or workshops)	109

^a^Museum attribution, not necessarily through signature; only inter-artwork comparisons considered.

### Interval between the production dates of signed and dated paintings with boards from the same tree

To estimate the amount of time between the completion of two paintings with boards from the same tree, we identified signed and dated artworks among those that met the statistical threshold. The same-tree origin of these boards was verified through visual comparison of the tree-ring graphs (Supplementary Materials_Graphs). There were only six pairs of paintings (12 paintings total) with boards from the same tree where both paintings were signed and dated by the artist(s) (Table 3). In three of those pairs, the paintings were signed and dated in the same year, while the remainders were signed and dated 1 or 2 years apart.

**Table 3:** Overview of panels with boards from the same tree, where both panels are signed and/or dated

Keycode A	Keycode B	T_BP_	Artwork A	Artwork B	Date A	Date B
4624001B	4565403B	22.8	Dirck Hals, *The Fête Champêtre*, Rijksmuseum SK-A-1796	Pieter de Grebber, *The Wrath of Ahasuerus*, Nationalmuseum NM 448	1627	1628
4009911 A	4001001 A	18.7	Rembrandt van Rijn, *Self-Portrait*, Private collection	Rembrandt van Rijn, *Portrait of Maurits Huygens*, Hamburger Kunsthalle 89	1632	1632
3004304A-C	3004303A-C	14.9-10.4	Peter Paul Rubens, Portrait of a Man, possibly Peter van Hecke (1591-1645), Mauritshuis 1131	Peter Paul Rubens, Portrait of a Woman, possibly Clara Fourment (1593-1643), Mauritshuis 1132	1630	1630
4705701A-B	4584003A-B	13.2-10.4	Salomon van Ruysdael, *River Landscape with a Ferry-Boat*, Princely Collections Liechtenstein GE 2510	Claude de Jongh*, Landscape*, Rijksmuseum SK-A-2842	1631	1633
4004307B	4004009 A	10.2	Rembrandt van Rijn, *Simeon’s Song of Praise*, Mauritshuis 145	Rembrandt van Rijn, Old Woman Reading, Probably the Prophetess Anna, Rijksmuseum SK-A-3066	1631	1631
4994071B	4994071B	10	Anonymous, Portrait of a Woman, possibly Elsje van Houweningen, Rijksmuseum SK-A-584	Anonymous, Portrait of a Man, presumably Willem van Velden, Rijksmuseum SK-A-583	1656	1657

Keycodes identify the matching boards (letters indicate individual boards within the panel; e.g. A-C indicates boards A, B and C). T_BP_ refers to the Baillie-Pilcher t-value^20^; where multiple boards meet the threshold, values are given as a range from highest to lowest. Dates A and B indicate the signed dates of the respective paintings.

### Interval between the production dates of art historically dated paintings with boards from the same tree

**Fig. 4:**
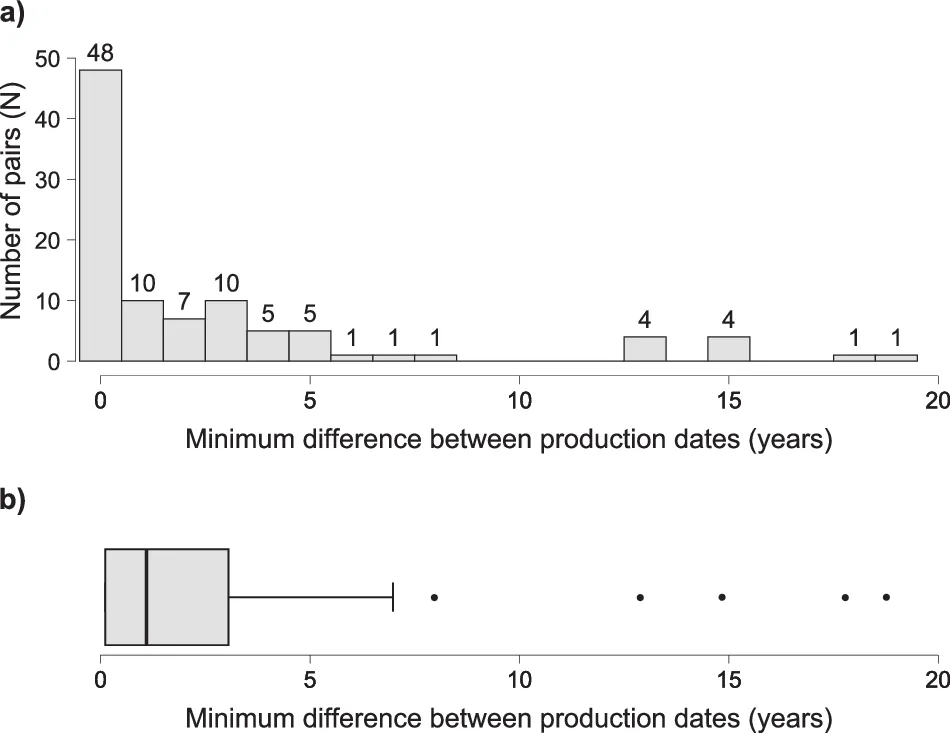
Time spanned between the production dates of signed and dated paintings with wood from the same tree. **a** Frequency histogram of the minimum interval between the production dates by year and **b** Box-Whisker plot of the minimum interval between the production dates in years, with the median (black), and suspected outliers (dots).

When a painting is not signed and/or dated by the artist, art historians can sometimes approximate the production date through secondary evidence, such as contracts, letters, or inventories. Including paintings for which an approximate production date has been determined based on art historical evidence, there are 98 pairs of paintings were both artworks are either dated by the artist, or the approximate production date is known (Supplementary materials_Data). Given the minimum interval between these approximations, this results in the distributions in Fig. 4.

The distribution of the minimum interval between approximate production dates clearly skews towards the right (skewness 2.1), with a median of 1 and a mean of 2.75. If the minimum interval between the estimated production dates of these paintings is presumed, approximately half (*N* = 48, 49%) of the paintings could have been completed within the same year, and around 87% (*N* = 85) of paintings were produced within a 5-year interval (Fig. 4). Given this pronounced skewness, intervals exceeding 10 years were treated as outliers corresponding to the upper tail (above the 90^th^ percentile). These observations likely represent exceptional cases (e.g., long-term storage, reuse of older panels, or uncertainties in dating) rather than typical practice. After excluding these values, the 95% confidence interval for the difference between production years for panel paintings with boards from the same tree is 1 to 1.8 (1.4 ± 0.4) years.

## Discussion

Although the signing and dating of paintings became more common in the Low Countries during the 17^th^ century, signing practices varied widely between artists^21^. As a result, most 17^th^-century paintings do not bear a signature or date, while in other cases, signatures may no longer be visible to the naked eye, or have been lost over time due to damage or cropping of panels. These circumstances explain why, despite the size of the initial dataset, the number of researched paintings that are dated by the artist is very limited. In addition, dendrochronological research is most often applied in cases where the creation date of the painting is unknown, which is quite unfortunate, because, as shown here, dendrochronological research on dated paintings sheds light into trade history and workshop practices.

In 1981, Bauch and Eckstein estimated an average of 5 ± 3 years for the interval between the felling of the tree and the production of panel paintings, while studies from the 1990s suggested intervals of 2–5 years^1,2^. Notably, the estimated interval for the 16^th^ and 17^th^ century is shorter compared to the late Middle Ages (8–10 years)^2,5^, indicating a faster rate of timber consumption during the more recent period.

Our data analysis suggests, however, that there may be a meaningful variation to this interval based on the provenance of the wood. Revising the provenance of the boards with sapwood with reference chronologies that were not available at the time of Klein’s research reveals that the boards can be broadly divided into three main provenances: (1) eastern Baltic, (2) Polish and (3) Eastern France/Belgium/Western Germany. These findings align with those of Fraiture^9^ and Seim et al.^4^, who note the reintroduction of the western Central European provenance by the end of the 16^th^ century in Flemish panels, marking a shift from the nearly-exclusive Polish-Baltic provenance in earlier centuries^4,9^. There is also another notable shift around 1650 (as previously observed by Jansma et al.^22^), after which the Polish-Baltic provenance suddenly disappears almost entirely (see Fig. 2)^3^.

The observed difference in the intervals between felling and use for Eastern Baltic/Polish versus western Central European provenance (Table 1) may be explained through several, not mutually exclusive, hypotheses that warrant further research:

- **Transportation time**: Timber sourced from the Baltic region required transport by ship from Baltic harbors to the Low Countries^8^, plausibly adding time to the period between the felling of the tree and the use of the wood compared to more locally sourced wood. In addition, the Baltic trade was occasionally interrupted by instability around the Sound Straight. For example, the Dutch-Spanish and Polish-Swedish wars repeatedly disrupted the import of Baltic timber to the Low Countries from the late 16^th^ century onwards^23^, leading to the search for alternative sources^4^, and increasing the time for timber to arrive in Dutch and Flemish ports.
- **Differences in seasoning time**: Different regions may have used different standards regarding the seasoning time required for oak. Archival records from Gdansk, for example, suggest that various types of oak products were sorted and left to dry in dedicated yards at the harbor for several months before they would be exported^24^. It is unclear whether other regions had similar practices.
- **Technological development**: Advances in woodworking technology, most notably the introduction of the wind-driven sawmill in the Low Countries^10^, may have accelerated the processing of timber, thereby shortening the overall time between the felling of the tree and the use of the wood.
- **Chronological shift**: The western Central European provenance is more prevalent in the second half of the 17^th^ century, and may therefore simply reflect an overall more rapid consumption of timber resources (possibly due to shortages caused by the loss of Baltic import).

**Fig. 5:**
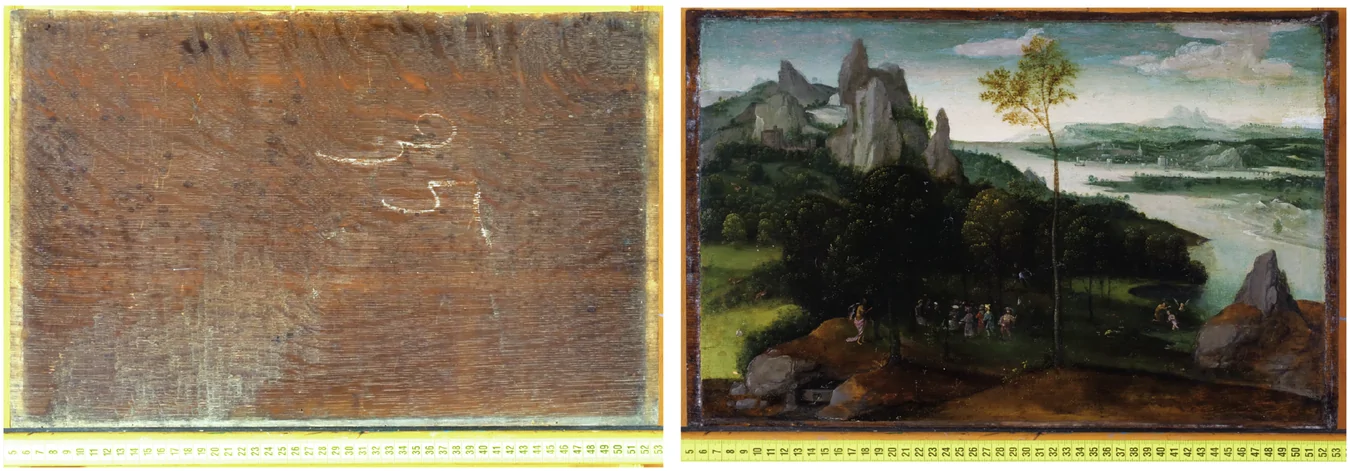
Example of a sapwood inclusion. Sapwood inclusions (light-colored) running horizontally in the board of a painting depicting a ‘Landscape with biblical scenery’ attributed to a follower of Joachim Patinir (Bouvignes-sur-Meuse, c. 1480 – Antwerp, 1524) (private collection, the Netherlands). Photos: Manon Engels.

When a long time-span has been calculated between the felling of the tree and the date in the signature, two possibilities arise. One could be the occurrence of sapwood inclusions (‘moon-rings’), which results in a false estimate of the felling of the tree that predates the true felling date. A sapwood inclusion is a light-colored zone made up by several sapwood rings remaining in the middle of the heartwood of oak trees (Fig. 5). They can be the result of severe frost or damage to the tree^25^. Its resemblance to true sapwood, especially when found along the edge of a board, may lead to the conclusion that the outermost part of the board was close to the bark, resulting in an estimated felling date of the tree (and an inferred production date of the painting) much earlier than what it was in reality. So far, there is no reliable method to distinguish sapwood inclusions from true sapwood in such cases. Therefore, the occurrence of sapwood inclusions offers a plausible explanation for the outliers with >20 years difference between the felling date of the tree and the use of the panel, even after the dendrochronological dates were verified in the provenance analysis. It is also possible that the signatures and/or dates on some panels have been falsified or added at a later time, postdating the true production date of the painting.

The occurrence of boards from the same tree in panels attributed to the same artist or workshop supports the hypothesis that painters were able to acquire batches of panels for their studio. Though documentation of panel purchases is scarce, evidence from inventories indicates that by the 17^th^ century, panel makers produced panels in a range of standardized sizes, enabling painters to purchase multiple standard-format supports at once^11^. This practice of batch-purchasing would explain why panels with boards from the same tree are regularly found in paintings attributed to the same artist or studio (Table 2). These findings replicate early conclusions by Bauch and Eckstein, who found that boards from the same tree occur frequently in the oeuvre of Rembrandt van Rijn^1^. Based on the observations of the interval between the production dates of signed and dated paintings with boards from the same tree, it is plausible that surplus panels were kept in stock in the studio for brief durations.

Several of the groups with boards from the same tree in the dataset are pendant portraits or companion pieces, for which a similar production date is expected. Previous research has shown that pendants on canvas regularly derive from the same bolt, indicating they were stretched and prepared simultaneously^26,27^. It can be assumed that panels for pendants were similarly purchased and prepared together, which explains the high occurrence of boards from the same tree in pendant pairs. The dataset furthermore includes several paintings from the same series, such as Rubens’ *Marie de’ Medici* cycle sketches and van Dyck’s Apostle series, for which supports were similarly likely purchased together or prepared on commission.

Boards from the same tree were also identified in paintings attributed to two or more different painters or workshops (Table 1). This suggests that the artists obtained the panels from the same source, likely a panel maker. A previous study by Wadum *et al*. examined a group of 17^th^-century panel paintings marked with the same panel-maker’s mark known as “4MM” (implying that the panels were produced in the same panel maker’s workshop)^10^. A set of three of these paintings, attributed to different artists, were found to contain boards from the same tree. Given that two of the paintings are signed and dated in 1640, the authors suggest that the remaining undated painting was likely produced in that same year. Our results support this hypothesis. Assuming that panels that contain boards from the same tree were produced and sold more or less contemporaneously, the time between the completion of the paintings on these supports therefore provides an indication of the rate at which the panels were used in 17^th^-century workshops.

In general, the short interval between panel paintings with boards from the same tree implies that panels were consumed at a high rate for artistic production. This aligns with the broader view of the prolific artistic output in the 17^th^-century Low Countries. Differences in production rates between workshops were likely shaped by factors such as the number of assistants, degree of specialization, and the financial standing of each workshop. Larger workshops, like those of Rembrandt van Rijn and Peter Paul Rubens, are well-represented in the dataset and may have influenced these findings. However, the pattern of short production intervals persists even for paintings completed in different workshops, as seen in Dirk Hals’ *Fête Champêtre* and Pieter de Grebber’s *Wrath of Ahasuerus*, suggesting that the rapid use of panels was a broader trend.

The relationship between the second board of Dirk Hals’ *Fête Champêtre*, dated 1627 (only visible with IR reflectography), and the second board of Pieter de Grebber’s *Wrath of Ahasuerus*, dated 1628, was not yet identified in previous research, but visual comparison of the tree-ring series strongly suggests the boards may indeed derive from the same tree (Supplementary Information_Graphs). Though there is little historical evidence of interactions between the two painters, both artists lived and worked in Haarlem at the time the paintings were completed. This suggests it is plausible the panels were either obtained from the same panel maker, or possibly the same intermediate seller, like an art dealer^10^. It may be possible to identify the exact source in the future through further archival research on panel suppliers active in and around the region of Haarlem.

The outliers among the results of the estimated intervals between production dates (Fig. 4) can be attributed to methodological constraints. As demonstrated by Hillam and Groves, the T_BP_ > 10 threshold provides a practical but imperfect approach to identifying boards from the same tree: some intra-tree comparisons fall below this threshold, whereas some inter-tree comparisons may exceed it^28^. To mitigate this limitation, a visual comparison of the tree-ring series was conducted. However, given that measurements obtained with the handheld lens methodology are of lower resolution compared to more recent methodologies^29^, these assessments were not always conclusive without re-examining the original panels. In other cases, the agreed-upon art historical date through secondary evidence may not accurately reflect the true production date of the artwork in question, leading to a disproportionate gap in the production dates.

While legacy dendrochronological data provides an opportunity to study the larger trends and patterns of artistic wood use in the 17^th^-century Low Countries, the use of such datasets in general, and in particular of the Dendro4art one, warrants some caution. The recent replication study by Domínguez-Delmás explains how the methodological approach of dendrochronological research in the 1990s was vulnerable to errors in different stages in the process, potentially leading to faulty measurement series^29^. In addition, the conversion of the dataset from older to newer file formats for archival purposes^30^ may have resulted in artifacts or omissions in the metadata. This hinders the re-use of this data for research, as the metadata is crucial for connecting the tree-ring measurements to the researched object. Here, for example, a number of measurement series were discarded from the same-tree analysis due to suspected errors, and similarly, we could not replicate the date and provenance of 36 of the boards with sapwood, likely due to the presence of measurement errors in those series as well. The verification and correction of faulty measurements would require the re-examination of the paintings if accessibility to the artworks is granted by the owners. This would allow the revision and replication of the dendrochronological research, enhancing the transparency of the attribution of artworks within the art community.

Our data analysis of 51 signed and dated panel paintings containing boards with partial sapwood suggests that the interval between tree felling and the completion of a painting varied according to the provenance of the timber. While wood imported from the Eastern Baltic is associated with longer intervals, more locally sourced Western-European timber shows shorter timeframes. Overall, the average lapse between felling and use falls between 4.6 and 7.5 years after felling, within a 95% confidence interval. This timeframe encompasses the seasoning and transportation of the wood, the construction of the panel, and its storage by artists and/or intermediary sellers, and indicates that wood for artistic practice was consumed at a rapid rate. The observed provenance-based differences likely reflect differences in seasoning and transportation time.

The analysis of signed and dated panels containing boards that derive from the same tree provides insight in the practice batch-purchasing panels by artists. Out of the 4101 boards analyzed, large-scale internal crossdating identified over two hundred instances where boards from two different paintings derive from the same tree. Examination of signed and dated or art historically dated works reveals that paintings sharing boards from the same tree were typically produced within a near-contemporary timespan, most often within 1–1.8 years. This finding provides the first empirical evidence that panel stock was consumed at a rapid pace in 17^th^-century Dutch and Flemish workshops, both within and across artists’ studios. These results underscore the value of dendrochronological studies of signed and dated panel paintings, a category that is typically overlooked for this type of research. Expanding such research has the potential to refine our understanding of the rates of timber consumption and artistic production in the early modern Low Countries.

The results of this study have significant implications for both dendrochronological and art historical dating of panel paintings. Dendrochronologists can use the interval between the felling date and the use of the wood to better estimate the production date of an undated panel painting based on the provenance of the wood. The interval between the production dates of panel paintings that contain boards originating from the same tree also has consequences for existing art historical attributions and chronologies. In cases where an undated painting contains boards from the same tree as a dated work, the probability is very high that both were produced within the same year, or within a maximum interval of approximately 2 years. In cases where panels with boards from the same tree have been assigned production dates that differ by a longer period, the results presented here suggest that these timelines may warrant reconsideration. The identification of boards from the same tree in panel paintings therefore provides an additional means by which dendrochronological research can complement archival sources and stylistic analysis, aside from dating the wood itself. Beyond questions of dating, our results contribute to a broader understanding of timber supply and use in 17^th^-century art workshops in the Low Countries.

Although this research is based on limited observations due to shortcomings of the legacy dendrochronological data from the Dendro4Art database, gaps in art historical research and the limitations of using crossdating thresholds, it highlights the importance of the preservation of such datasets, and the value of computational approaches to analyze them. Future research combining large-scale dendrochronological data with art historical research is needed to explore how panel use varies across different time periods, geographic regions, timber provenances, opening the door to new perspectives on artistic production and material consumption in Early Modern Europe.

## Methods

### Dataset collection

The data used in this research derives from an archive of >6000 research reports and their associated 16,600 tree-ring width (TRW) series from individual boards created by dendrochronologist Peter Klein (Hamburg University, Germany). Over four decades, Klein collaborated with at least 220 institutions and private collectors to examine a variety of wooden art objects from German, Austrian, Flemish, Dutch, English, French, Italian, Spanish, Portuguese, Polish, Romanian, Hungarian, Finnish and Swedish collections. In 2013, Klein’s research archive was acquired and digitized by the Netherlands Institute for Art History (RKD) and the Danish Centre for Art Technological Studies and Conservation (CATS) as part of the Dendro4Art project (https://dendro4art.org/).

For the present study, a subset of TRW series was selected from this legacy dataset based on the following selection criteria:

1. The examined board was made of oak (*Quercus* sp.), designated in the metadata as QUSP. Any other species were excluded from this research.
2. Only keycodes starting with 3 or 4, representing attributions to Flemish and Dutch 17^th^-century painters respectively, were retained. However, as this classification is attribution-based, a small number of panels included in the dataset may in fact date to the late 16^th^- or early 18^th^-century.
3. The dataset was restricted to panel paintings; records relating to other object types such as frames or furniture were removed.
4. TRW series for which the date could not be replicated or with data-entry errors were excluded.

The resulting dataset comprises 4101 TRW series of oak (*Quercus* sp.) boards that derive from 1,991 different panel paintings attributed to >300 Dutch and Flemish 17^th^-century artists.

### Metadata extraction using R

For the purpose of this study, we divide the data into two types: primary data, consisting of the tree-ring width measurements, and metadata, referring to the descriptive data contained in the headers of each file, including the keycode (unique identifier of the tree-ring measurement for each board), the series’ length (number of tree rings), start and end year, location (in which Klein listed the attribution, inventory number, and title keywords at the time of research), species, and number of sapwood rings. The metadata of the files in the dataset was extracted using R (v. 4.3.1), an open-source programming language and software environment, with a modified version of the dplR package’s read.fh function and combined into a single data frame^31^.

### Identification of signed and dated paintings

The extracted metadata served as a starting point to identify artworks in the dataset to be selected for this research. The attribution (with or without signature) and the (approximate) production date was verified with museum catalog records (see Supplementary materials). Works held in private collections had to be excluded from the analysis due to the limited availability of public information. Paintings were considered to be “dated” if one or more of the following circumstances were met:

- The painting bears a date alongside the artist’s signature;
- The painting bears a contemporary inscription on the front or reverse with the date (such inscriptions are commonly found on portraits);
- There is secondary documentation, such as dated letters, contracts, or similar evidence that allows art historians to conclude the exact production time.

### Identification of boards with sapwood

The list of metadata was used to identify the boards in the dataset that retained (partial) sapwood. Then, Peter Klein’s research reports were consulted to verify the wood provenance. At the time of Klein’s research, there were only a few regional chronologies available. Therefore, he always described the provenance as either Polish/Baltic, or Netherlands/West-Germany. He then used the sapwood statistics proposed by Wazny to estimate the felling date of the trees when the wood was Polish/Baltic^8^, and those by Hollstein when the wood was of German/Netherlandish origin^16,19^. According to Wazny, trees in Poland had between 9 and 24 sapwood rings, with 15 being the median, whereas Hollstein reported that oaks had between 8 and 38 rings^5,16,19^.

### Revision of the provenance and estimation of felling dates

With sapwood observations from living trees being published during the 1990s and 2000s, it became obvious that the number of sapwood rings in oaks decreases in Europe along a west-east gradient^5^. Subsequently, Sohar *et al*. reported new sapwood statistics for oaks growing in the eastern Baltic, suggesting that they produce between 6 and 18 rings (within a 95% confidence interval)^6^. The number of tree-ring chronologies has also grown in the last decade. Therefore, we decided to revise the provenance of the boards with sapwood, and also the estimated felling dates accordingly.

Tree-ring series of boards with sapwood were compared with a set of chronologies representing the Baltic region^3^ and the northeast of France, Belgium and Germany (Meuse, Moselle, and Rhine river catchments)^16–18^, which were non-Baltic source areas supplying wood for panel painting in the Low Countries during the 17^th^ century^4,9^. This comparison was carried out in PAST4 v.4.3.1032^32^. The chronology providing the highest statistical match expressed by a T_BP_-value (Student’s *t*-value computed after applying the normalization algorithm proposed by Baillie and Pilcher^20^) was considered to represent the source area of the wood. In cases where there was no strong significant statistical match (T_BP_ < 5.0) the boards were excluded from the analyses (Supplementary Materials_Data).

Once the provenance was established as either eastern Baltic, Poland or northeastern-France/Belgium/Germany, we estimated the felling dates of the trees using the sapwood statistics corresponding to those areas: 6-18 for the eastern Baltic^6^, 9-24 for Poland^19^, and 8-38^5,16^ for the third group of sources. The calculation involved deducting the number of present sapwood rings in the board to the lower and upper number of the corresponding interval, and then adding the resulting numbers to the end date of the outermost (most recent) ring in the boards^5,6^. The calculations were performed in R using the fellingdater package^33^.

To create a balanced estimate of the interval between the felling of the tree and the production of the painting, we next calculated the median between the two dates and subtracted it from the signed production date of the painting. Any negative intervals were converted to 0, because the felling date of the tree cannot postdate the production date. The median, mean, standard deviation and 95% confidence interval by provenance were calculated using JASP (ver. 0.95.0), which was also used to create the graphs^34^.

### Internal crossdating using R

The identification of boards that derive from the same tree trunk is a crucial analysis in dendrochronology of panel paintings, as finding boards from the same tree in different artworks helps to narrow down the production dates of all of them through inferences, especially when sapwood is present in one of the boards, or even better, when one of the artworks is dated by the artist. Despite the limited capacity of dendrochronology software in the 20^th^ century, Klein made persistent efforts to identify boards originating from the same tree^35^. Currently, the Dendro4Art database registers approximately 1100 entries of boards from the same tree within the same artwork, and 600 cases of boards from the same tree that have been identified in two separate artworks within Klein’s legacy dataset.

During most of his research career, however, Klein worked within the constraints of the DOS program CATRAS^36^, which only allowed the comparison of one tree-ring series against a group of a limited size (more modern software such as PAST4^32^ is also limited to comparing a maximum of 2000 tree-ring series against each other at a time). These constraints force dendrochronologists to hand-pick which files to compare to identify boards from the same tree, introducing potential biases and overlooking potentially unexpected results. For example, new dendrochronological data obtained from a Rembrandt painting might firstly be compared to other panels from Rembrandt’s oeuvre, and subsequently, to a few of Rembrandt’s contemporaries, rather than all researched panels from the 17^th^-century Low Countries. Running the comparison on a larger dataset may result in new or unexpected matches that have previously been overlooked. With an ever-growing dataset of examined artworks, using tools that allow the comparison of large datasets is necessary.

To overcome this limitation, the internal crossdating of the dataset was conducted using R (v. 4.3.1), drawing upon existing R packages for dendrochronology. First, the selected TRW series (see Dataset collection) were compiled into a single data frame using the read.fh and combine.rwl functions from the dplR library^31^. Internal crossdating was done using the cor_table() function from the fellingdateR package, with a minimum overlap of 50 and Student’s *t-*value (according to Baillie and Pilcher^20^) threshold of > 10^33^. While the t-value > 10 threshold does not guarantee that two boards originate from the same tree^37^, it provides a valuable starting point to identify such groups in a dataset of this scale. To reach a same-tree conclusion, the match between pairs of tree-ring series surpassing this threshold was visually examined using PAST4 v.4.3.1025^32^. When in doubt, the boards were excluded from the analysis.

Combining the results of the internal crossdating analysis with the historical information described above (see Identification of signed and dated paintings), we compiled a list of boards originating from the same tree that belong to paintings signed and dated by the artist. In addition, matching groups of boards associated with paintings for which an estimated production date or interval could be established from secondary historical sources (e.g., letters or contracts) were included. For each group of artworks, the minimum interval between the estimated production dates was calculated on a pairwise basis (i.e., in cases where paintings A, B and C contain boards from the same tree, the minimum interval between the production dates of A and B, B and C, and A and C are included. In cases where paintings A and B have two or more boards from the same tree, the interval is only counted once). Descriptive statistics and the box-whisker plot and frequency histogram were calculated and created in JASP (ver. 0.95.0)^34^.

## Supplementary information

Supplementary materials - Graphs_revised

Supplementary materials - Data_revised

## Data Availability

The metadata of the paintings selected for this study has been made available through the Supplementary Information. The tree-ring dataset used in this study will soon be made openly available by the Netherlands Institute for Art History (RKD) through RKDTechnical, and meanwhile is available upon request to the RKD (technical@rkd.nl).

## Code availability

The R code used in the analysis derives directly from open-source R packages: the dplR package by Andrew Bunn (2010) and the fellingdateR package by Kristoff Haneca (2024).

## References

[1] Bauch, J. & Eckstein, D. Woodbiological investigations on panels of Rembrandt paintings. *Wood Sci. Technol.* **15**, 251–263 (1981).

[2] Wadum, J. Historical overview of panel-making techniques in the northern countries. in *The Structural Conservation of Panel Paintings* (eds Dardes, K. & Rothe, A) 149–177 (1998).

[3] Daly, A. & Tyers, I. The sources of Baltic oak. *J. Archaeol. Sci.* **139**, 105550 (2022).

[4] Seim, A. et al. Timber trade in 17th-century Europe: different wood sources for artworks of Flemish painters. *Sci. Rep.* **14**, 18216 (2024). 10.1038/s41598-024-68641-y PMC11303553 39107393

[5] Haneca, K., Čufar, K. & Beeckman, H. Oaks, tree-rings and wooden cultural heritage: a review of the main characteristics and applications of oak dendrochronology in Europe. *J. Archaeol. Sci.* **36**, 1–11 (2009).

[6] Sohar, K., Vitas, A. & Läänelaid, A. Sapwood estimates of pedunculate oak (*Quercus robur* L.) in eastern Baltic. *Dendrochronologia* **30**, 49–56 (2012).

[7] Eckstein, D., Wazny, T., Bauch, J. & Klein, P. New evidence for the dendrochronological dating of Netherlandish paintings. *Nature* **320**, 465–466 (1986).

[8] Wazny, T. The origin, assortments and transport of Baltic timber. in *Constructing Wooden Images: Proc. Symposium on the Organization of Labour and Working Practices of Late Gothic Carved Altarpieces in the Low Countries* (eds van de Velde, C., Beeckman, H., van Acker, J. & Verhaeghe, F.) 115–126 (Brussels University Press, 2005).

[9] Fraiture, P. Contribution of dendrochronology to understanding of wood procurement sources for panel paintings in the former Southern Netherlands from 1450 AD to 1650 AD. *Dendrochronologia* **27**, 95–111 (2009).

[10] Wadum, J., Domínguez-Delmás, M. & Jager, A. Unraveling a 17th-Century North Netherlandish Panel Maker. *Int. J. Wood Cult*. 277–304, 10.1163/27723194-bja10013 (2022).

[11] Bruyn, J. Een onderzoek naar 17de-eeuwse schilderijformaten, voornamelijk in Noord-Nederland. *Oud Holl. Art. Low. Ctries.* **93**, 96–113 (1979).

[12] Kleinert, K. *Atelierdarstellungen in der niederländischen Genremalerei des 17. Jahrhunderts: realistisches Abbild oder glaubwürdiger Schein?* (Michael Imhof, Petersburg, 2006).

[13] Bredius, A. Künstler-Inventare; Urkunden zur Geschichte der holländischen Kunst des 16ten, 17ten und 18ten Jahrhunderts. (M. Nijhoff, 1915).

[14] Bauch, J., Eckstein, D. & Meier-Siem, M. Dating the Wood of Panels by a Dendrochronological Analysis of the Tree-Rings. *Ned. Kunsthist. Jaarb*. *NKJ Neth. Yearb. Hist. Art.* **23**, 485–496 (1972).

[15] Klein, P., Eckstein, D., Wazny, T. & Bauch, J. New Findings for the Dendrochronological Dating of Panel Paintings of the 15th to 17th Century. In *ICOM Committee for Conservation 8th Triennial Meeting Sydney Australia 6-11**September 1987* (ed. Grimstad, K.) 51–54 (Getty Conservation Institute, Sydney, 1987).

[16] Hollstein, E. *Mitteleuropäische Eichenchronologie*. (Philipp von Zabern, Mainz am Rhein, 1980).

[17] Hoffsummer, P. *Les Charpentes de Toitures En Wallonie*. (Ministère de la Région Wallonne, Division du Patrimoine, Namur, 1999).

[18] Tegel, W., Vanmoerkerke, J. & Büntgen, U. Updating historical tree-ring records for climate reconstruction. *Quat. Sci. Rev.* **29**, 1957–1959 (2010).

[19] Wazny, T. Aufbau und Anwendung der Dendrochronologie für Eichenholz in Polen. (Hamburg University, Hamburg, 1990).

[20] Baillie, M. G. L. & Pilcher, J. R. A Simple Crossdating Program for Tree-Ring Research. *Tree-Ring Bull.* **33**, 7–14 (1973).

[21] Franken, M. & van der Veen, J. The Signing of Paintings by Rembrandt and His Contemporaries. In *The Leiden Collection Catalogue* (eds Wheelock Jr., A. K., Nogrady, E. & Van Cauwenberge, C.) 2–18 (The Leiden Collection, New York, 2022).

[22] Jansma, E., Hanraets, E. & Vernimmen, T. Tree-ring research on Dutch and Flemish art and furniture. *TRACE-Tree Rings*. *Archaeol. Climatol. Ecol.* **2**, 139–146 (2004).

[23] Tossavainen, J. Dutch forest products’ trade in the Baltic from the Late Middle Ages to the Peace of Munster in 1648. (University of Jyväskylä, Jyväskylä, 1994).

[24] Rief, M. Engraved Marks on Baltic Wainscot Boards. In *Constructing Wooden Images: Proc. Symposium on the Organization of Labour and Working Practices of Late Gothic Carved Altarpieces in the Low Countries* (eds van de Velde, C., Beeckman, H., Van Acker, J. & Verhaeghe, F.) 127–146 (Brussels University Press, 2005).

[25] Dujesiefken, D., Liese, W. & Bauch, J. Discolouration in the Heartwood of Oak-Trees. *IAWA J.* **5**, 128–132 (1984).

[26] Wetering, E. van de. The Canvas Support. in *A Corpus of Rembrandt Paintings II: 1631-1634* vol. 2 15–44 (Martinus Nijhoff Publishers, 1986).

[27] Johnson Jr, C. R. & Sethares, W. A. Canvas Weave Match Supports Designation of Vermeer’s Geographer and Astronomer as a Pendant Pair. *J. Hist. Netherlandish Art* **9**, 1–9 (2017).

[28] Hillam, J. & Groves, C. Tree ring research at Windsor Castle: aims and initial results. *Tree Rings Environ. Humanit. Radiocarb. Tucson* **515**, 524 (1996).

[29] Domínguez-Delmás, M. A replication study in dendrochronology—revisiting the panels of two portraits of Rembrandt. *Humanit. Soc. Sci. Commun.* **12**, 1778 (2025). 10.1057/s41599-025-06066-2 PMC12629980 41281721

[30] RKD Netherlands Institute for Art History. About Dendro4Art. Dendro4art https://dendro4art.org/about.html.

[31] Bunn, A. G. A dendrochronology program library in R (dplR). *Dendrochronologia* **26**, 115–124 (2008).

[32] Knibbe, B. PAST4—Personal Analysis System for Treering Research (Version 4.3.1025) [Computer software]. Scientific Engineering & Manufacturing (SCIEM), Vienna http://www.sciem.com/ (2009).

[33] Haneca, K. fellingdater: a toolkit to estimate, report and combine felling dates derived from historical tree-ring series. *J. Open Source Softw.* **9**, 6716 (2024).

[34] JASP Team. JASP (Version 0.95.0) [Computer software]. University of Amsterdam, Amsterdam. https://jasp-stats.org/ (2025).

[35] Klein, P. Dendrochronological Analyses of Netherlandish Paintings. In *Recent Developments in the Technical Examination of Early Netherlandish Painting: Methodology, Limitations & Perspectives* (eds Faries, M. & Spronk, R.) 65–81 (Brepols Publishers, Turnhout, 2003).

[36] Aniol, R. Tree-ring analysis using CATRAS. *Dendrochronologia* **1**, 45–53 (1983).

[37] Bernabei, M. Is a T-test value > 10 really reliable in identifying wood from the same tree trunk? *Dendrochronologia* **76**, 126025 (2022).

