# Supplementary material for: Legacy tree-ring dataset reveals rapid use of panels in 17th-century Dutch and Flemish painting workshops
Source: NPJ Herit Sci. 2026 Jun 24;14(1):362. doi: 10.1038/s40494-026-02737-8 (PMC13372665; doi:10.1038/s40494-026-02737-8)
Supplement: Supplementary file 1 — Supplementary materials - Graphs_revised [file 40494_2026_2737_MOESM1_ESM.docx]

# SUPPLEMENTARY MATERIALS – GRAPHS

# Pairs of boards^[[1]](#footnote-1)^

## 1


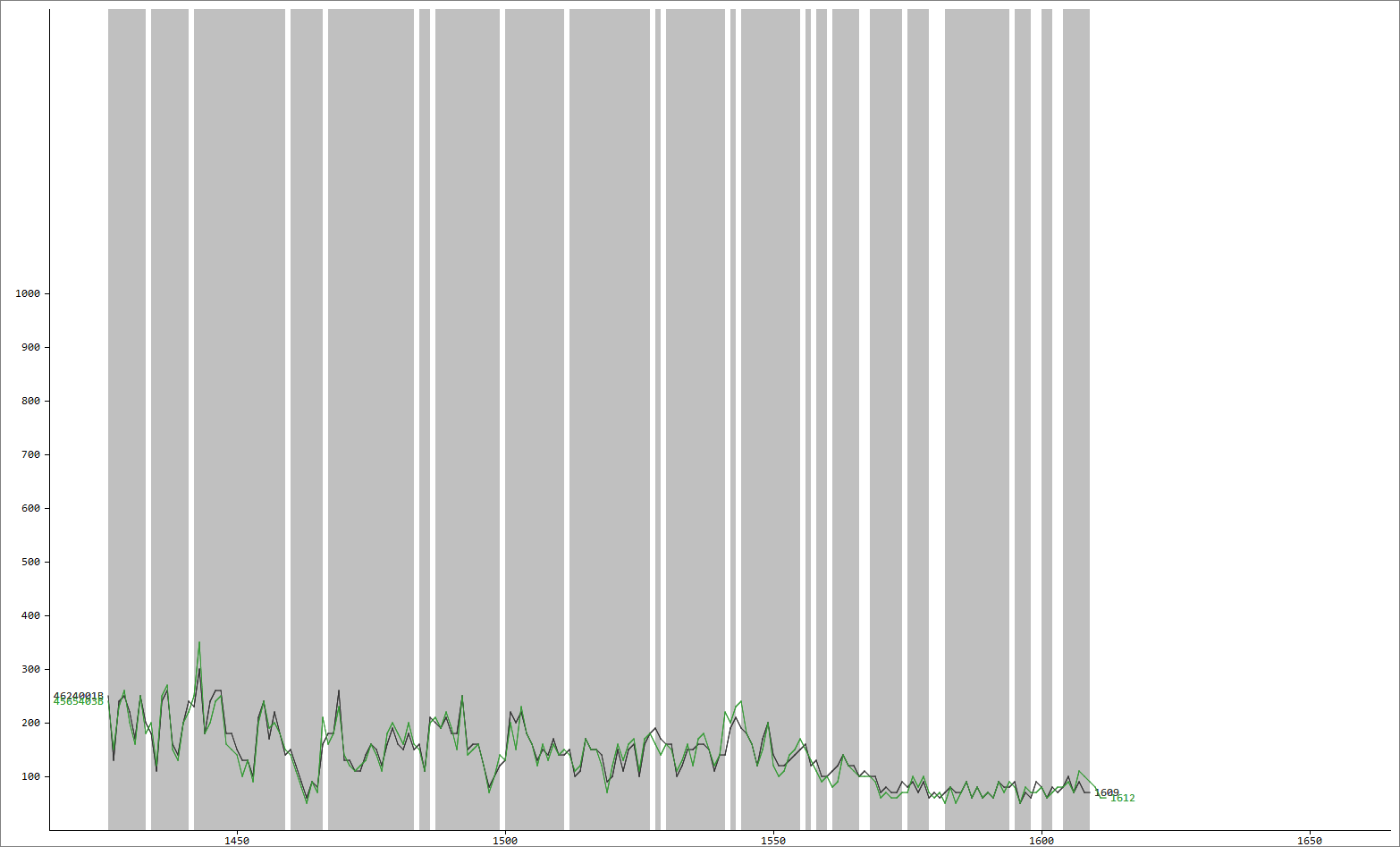


## 2

NB: The following graphs correspond to four boards from two paintings, where one board from one paintings matches a board from the other, and the same with the other two boards. As such, the graphs are presented together.


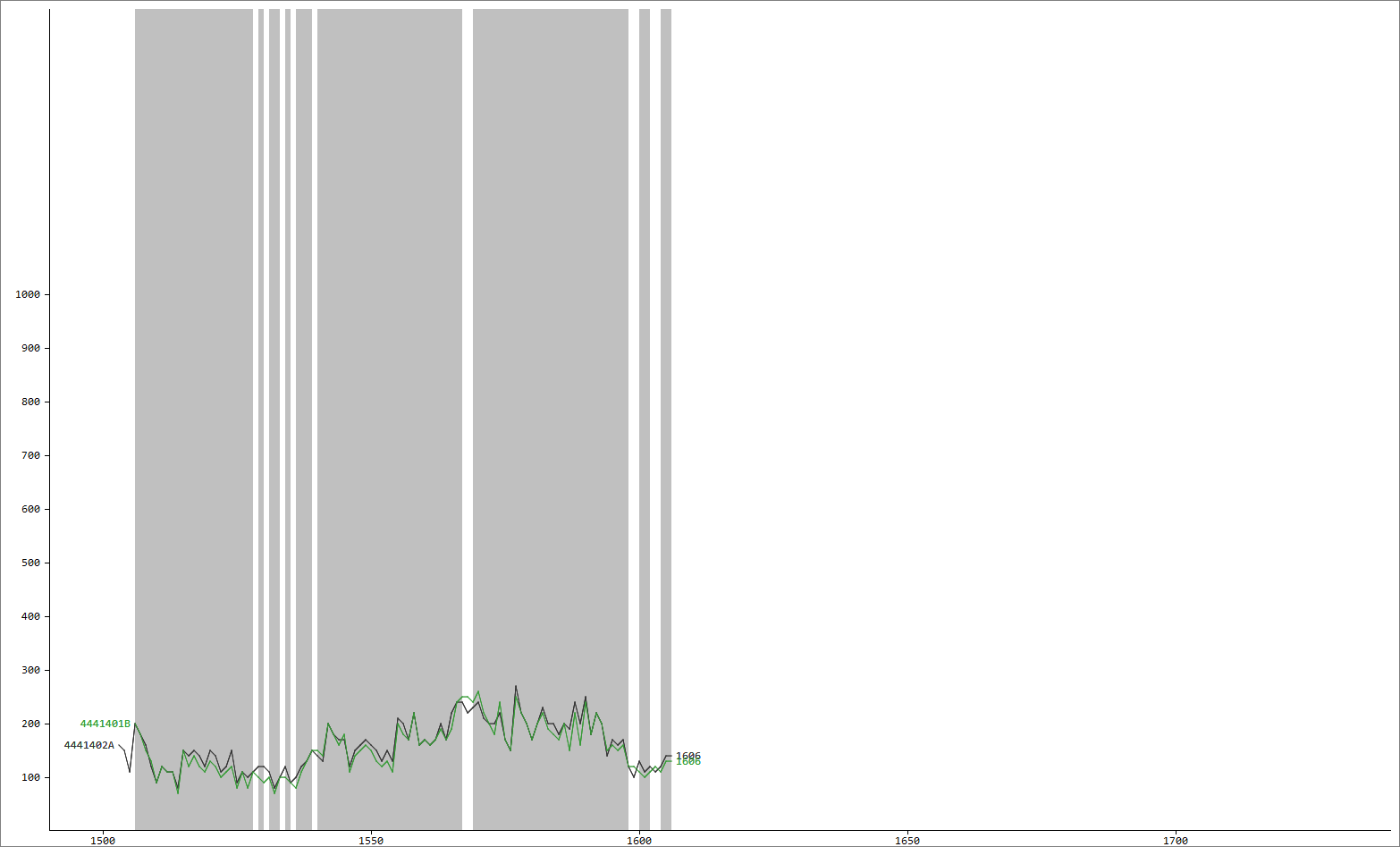


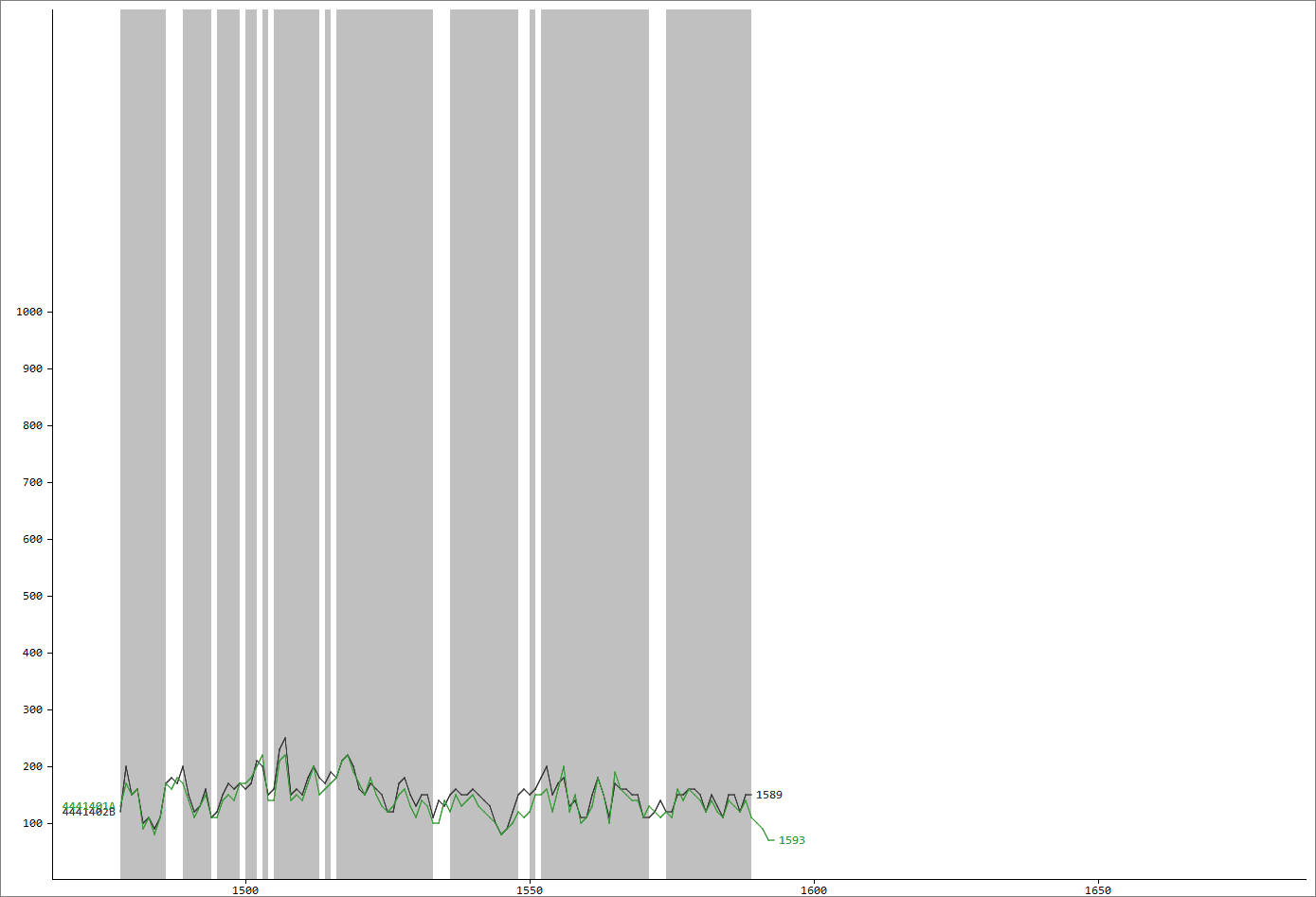


## 3


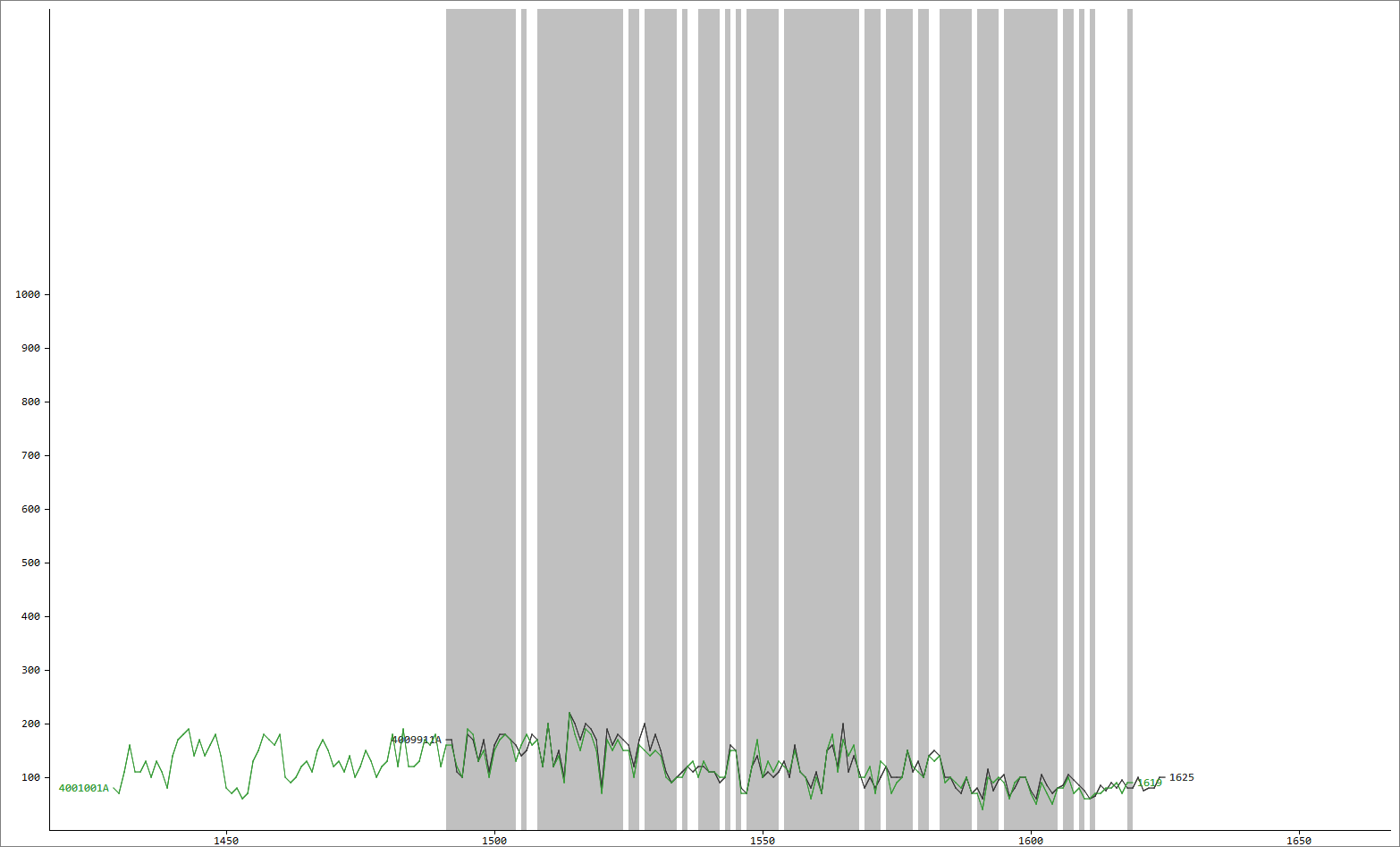


## 4


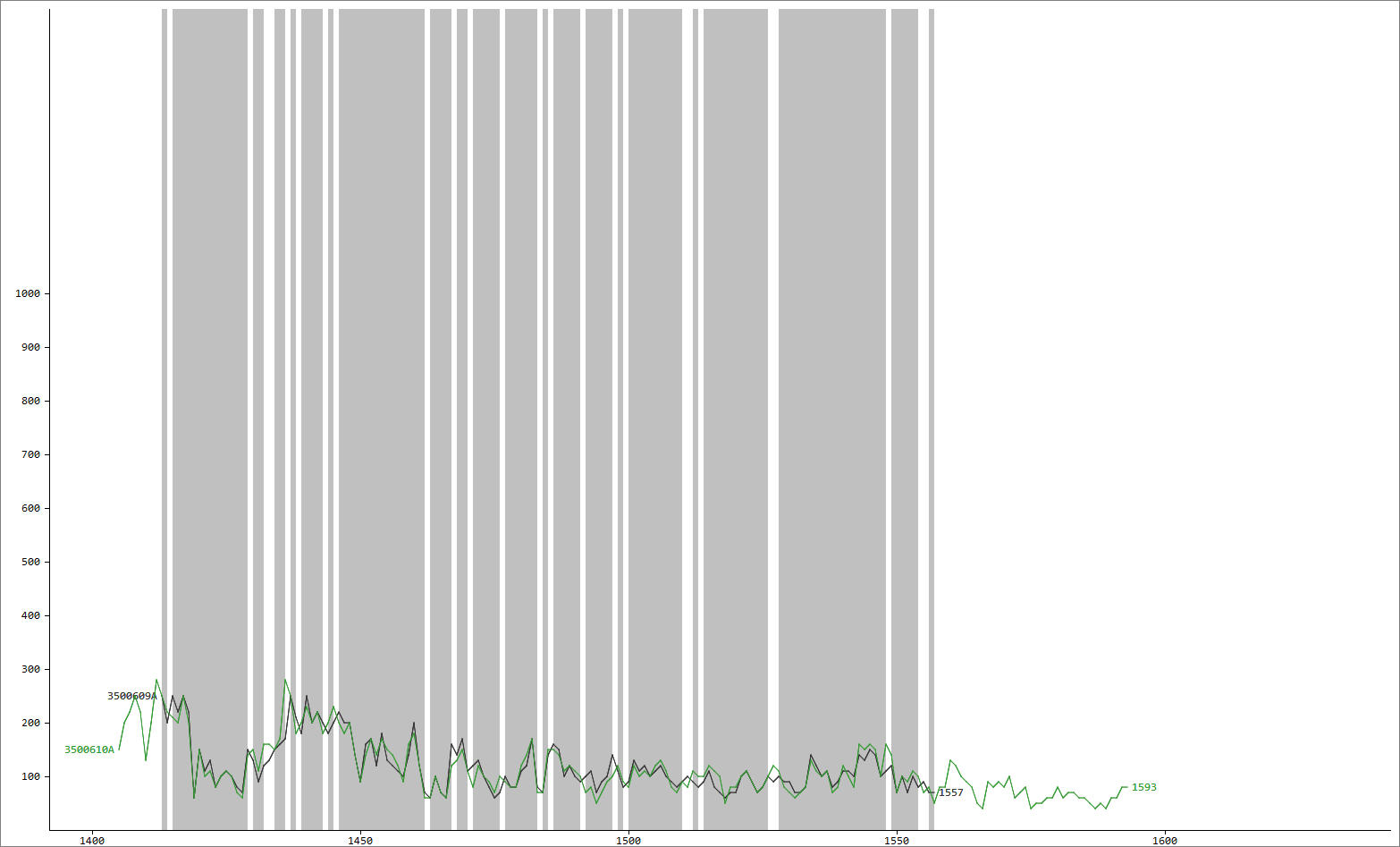


## 5


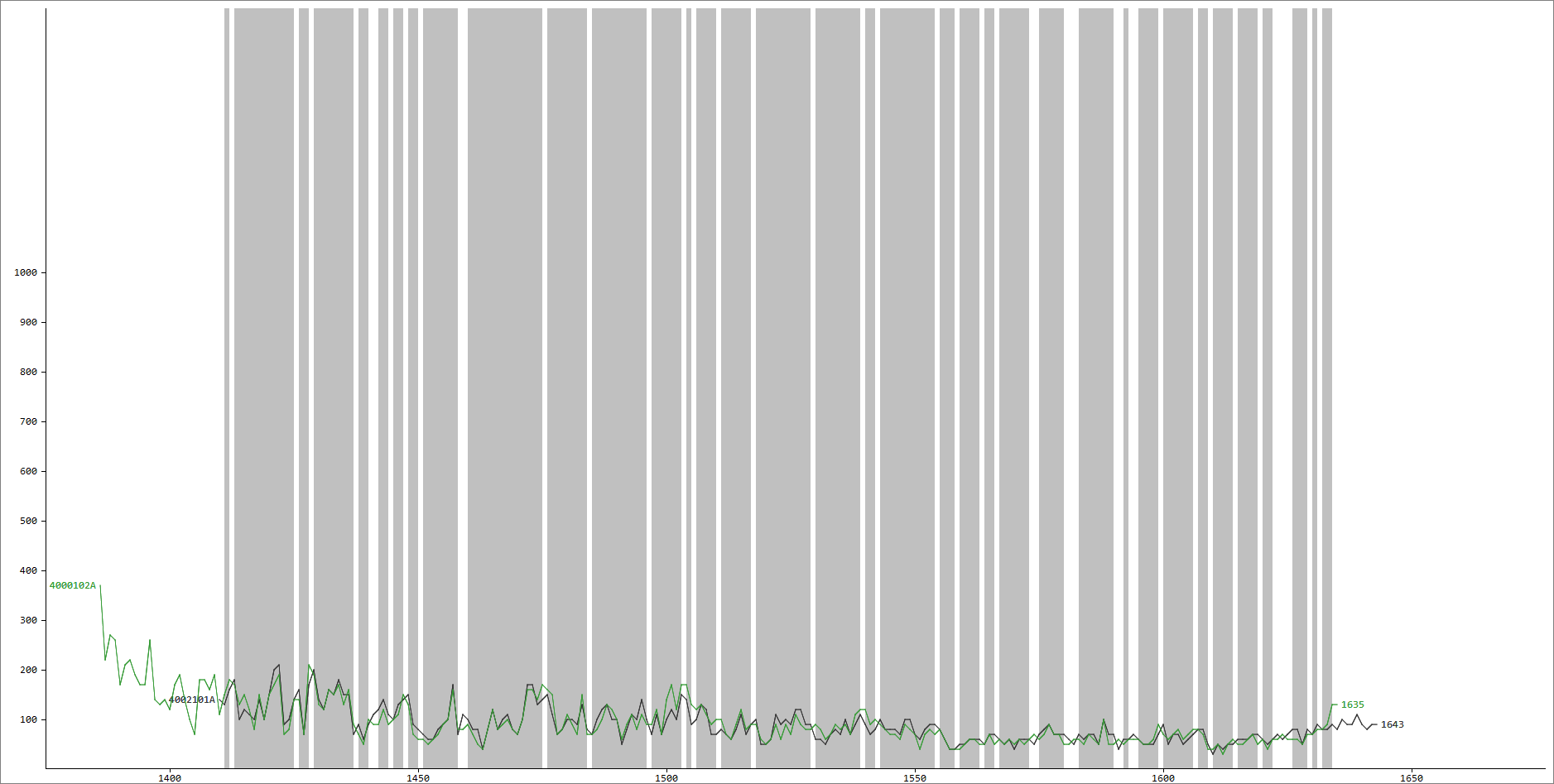


## 6


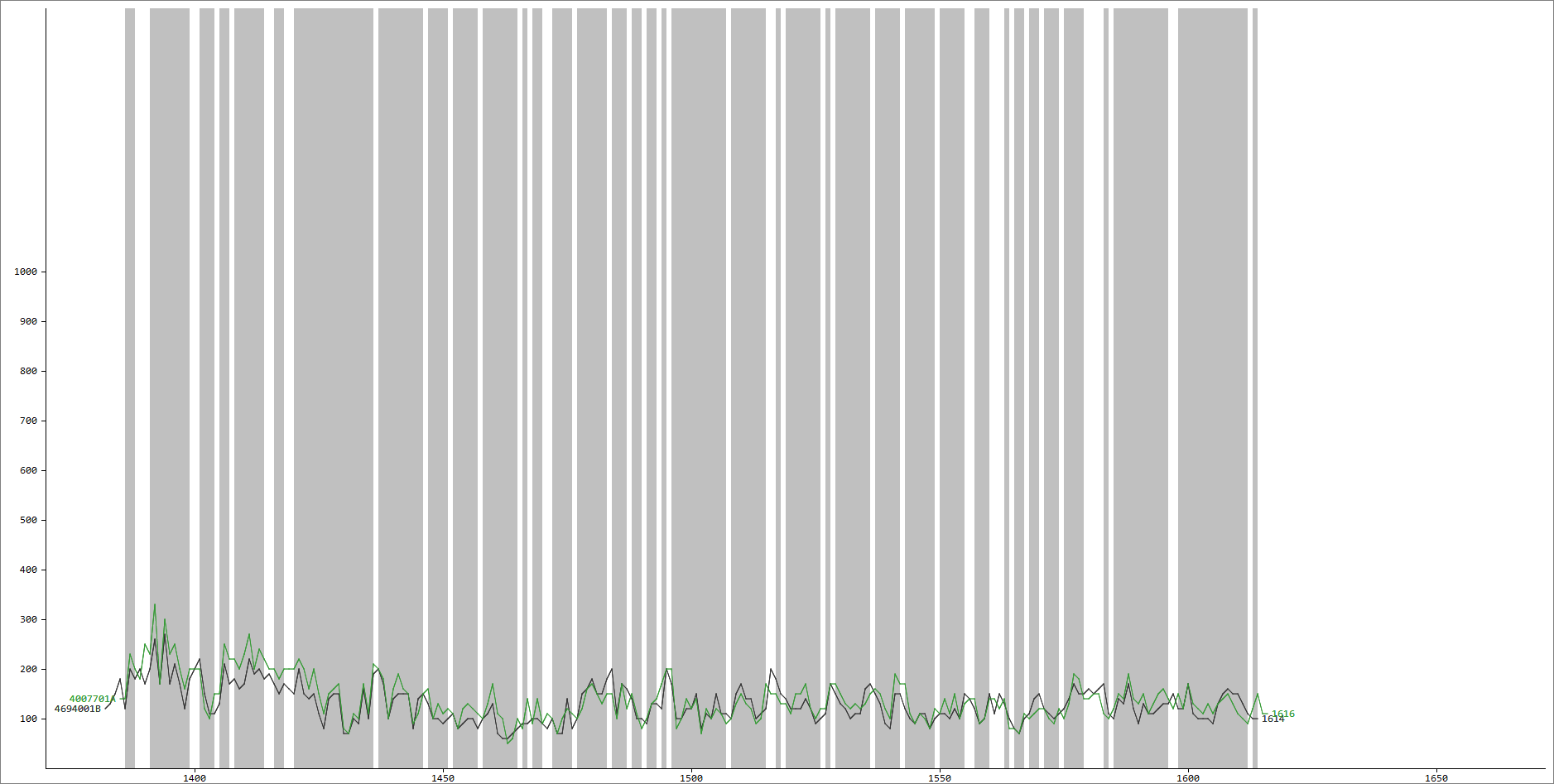


## 7


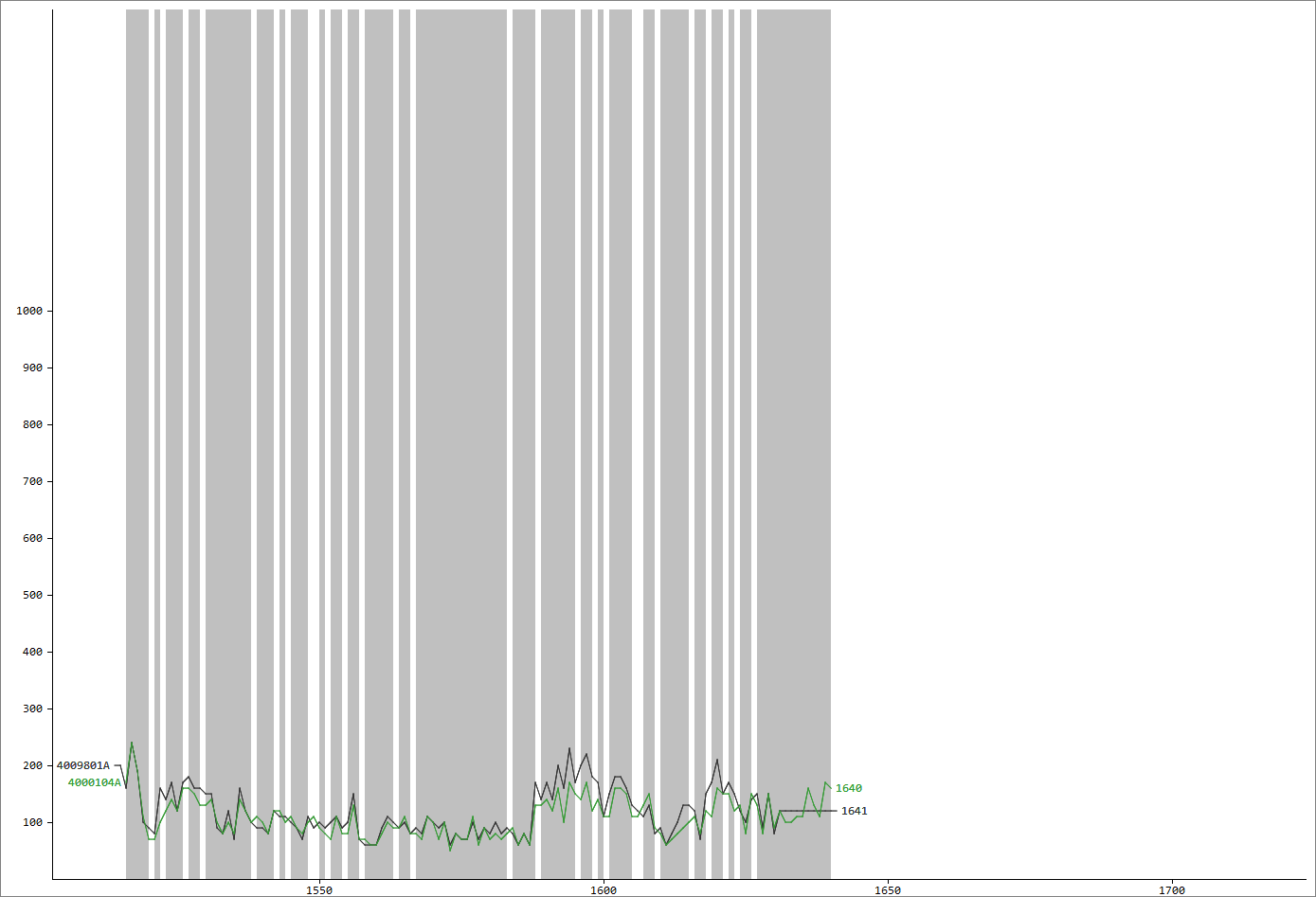


## 8


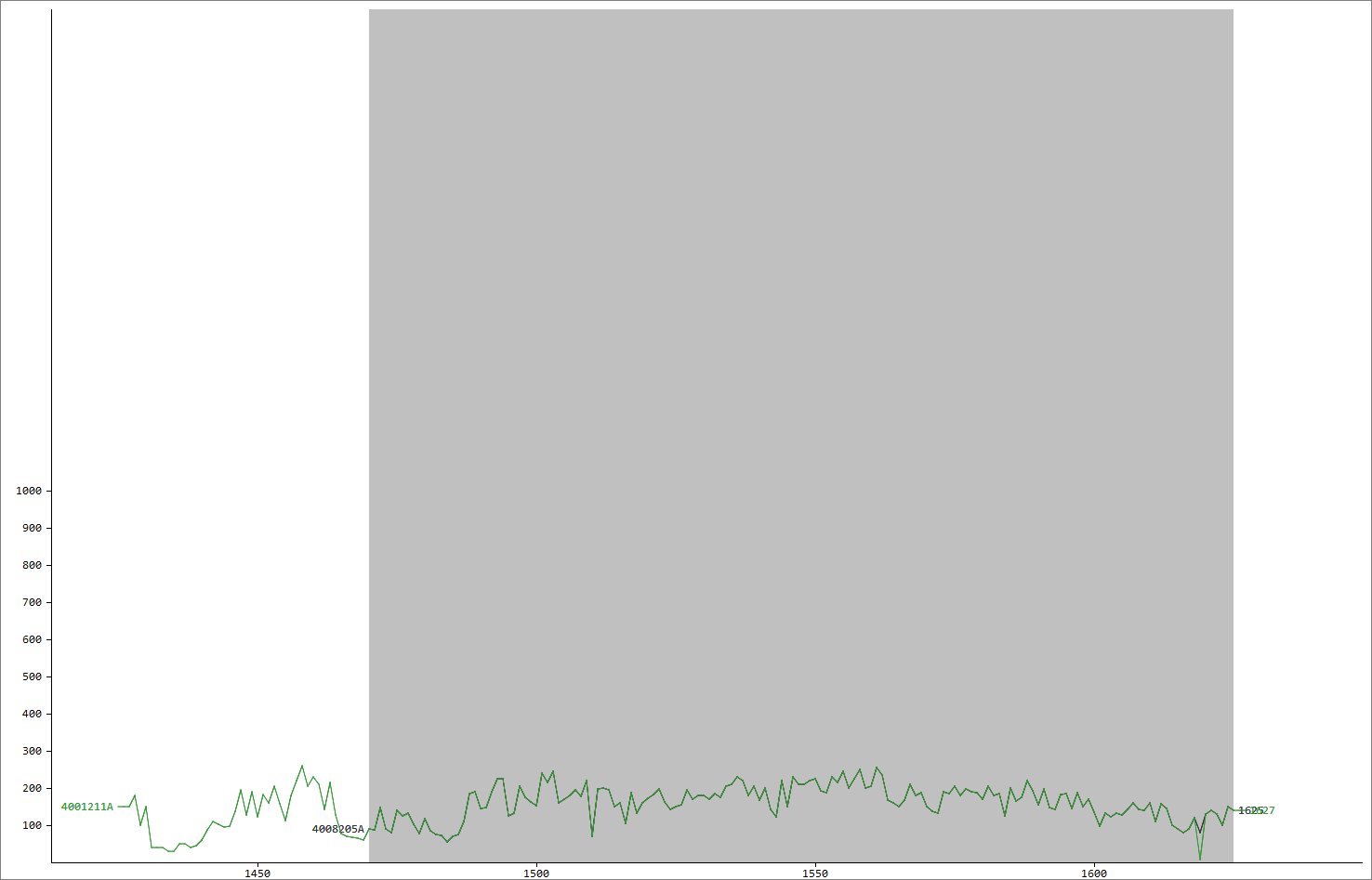


NB: The tree-ring series 4008205A and 4001211A are nearly identical, which is atypical even for boards from the same tree and may indicate a (partial) duplication in the original data transcription. As these measurements derive from a legacy dataset and cannot be verified, the same-tree association was retained in the context of this study per previously published results ([Klein 1992](https://rkd.nl/technical/5008958); [Klein 1995](https://rkd.nl/technical/5008497); Corpus of Rembrandt Paintings, vol. III), but should be treated with caution pending future re-examination of the boards.

## 9


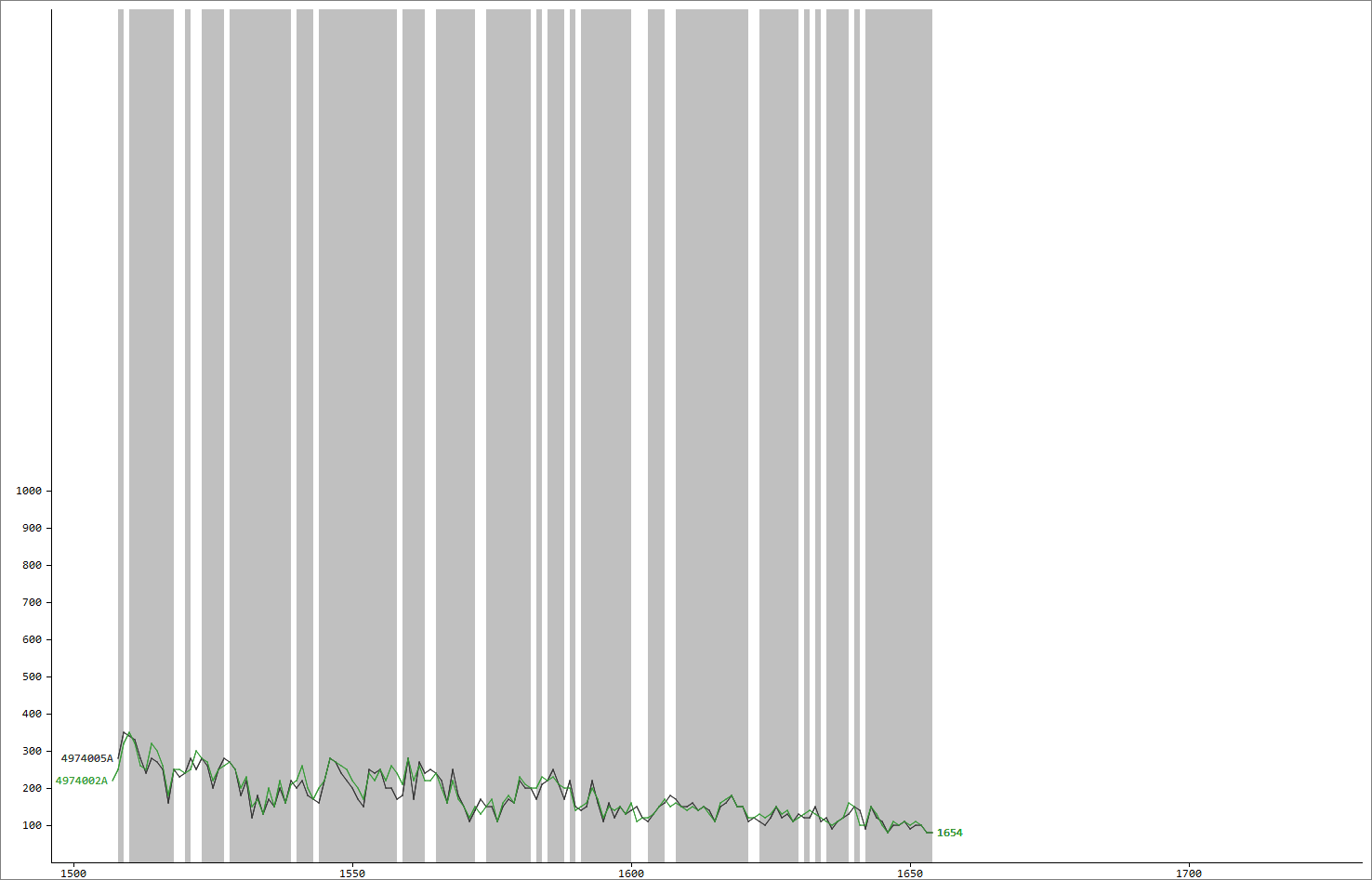


## 10


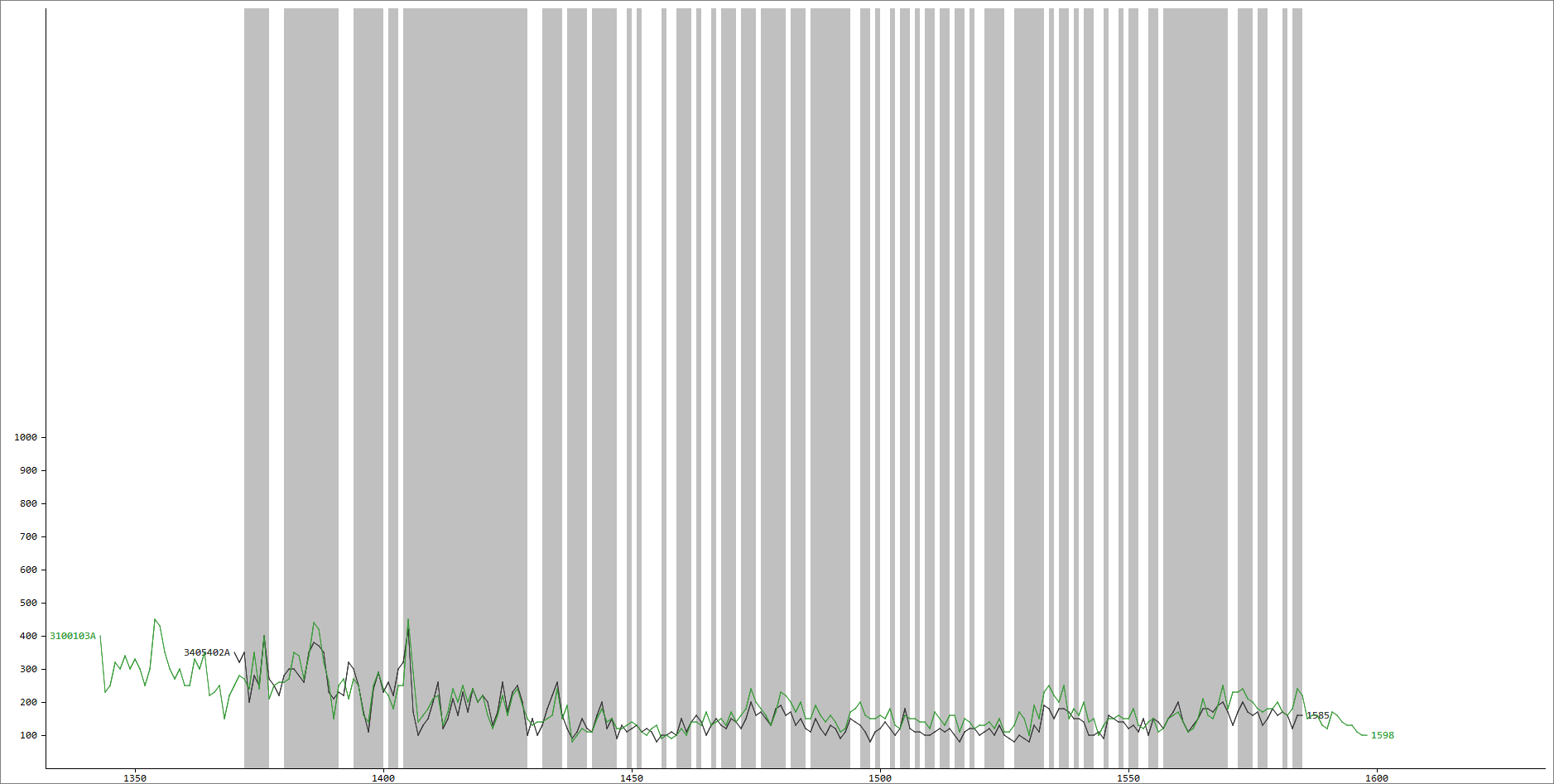


## 11


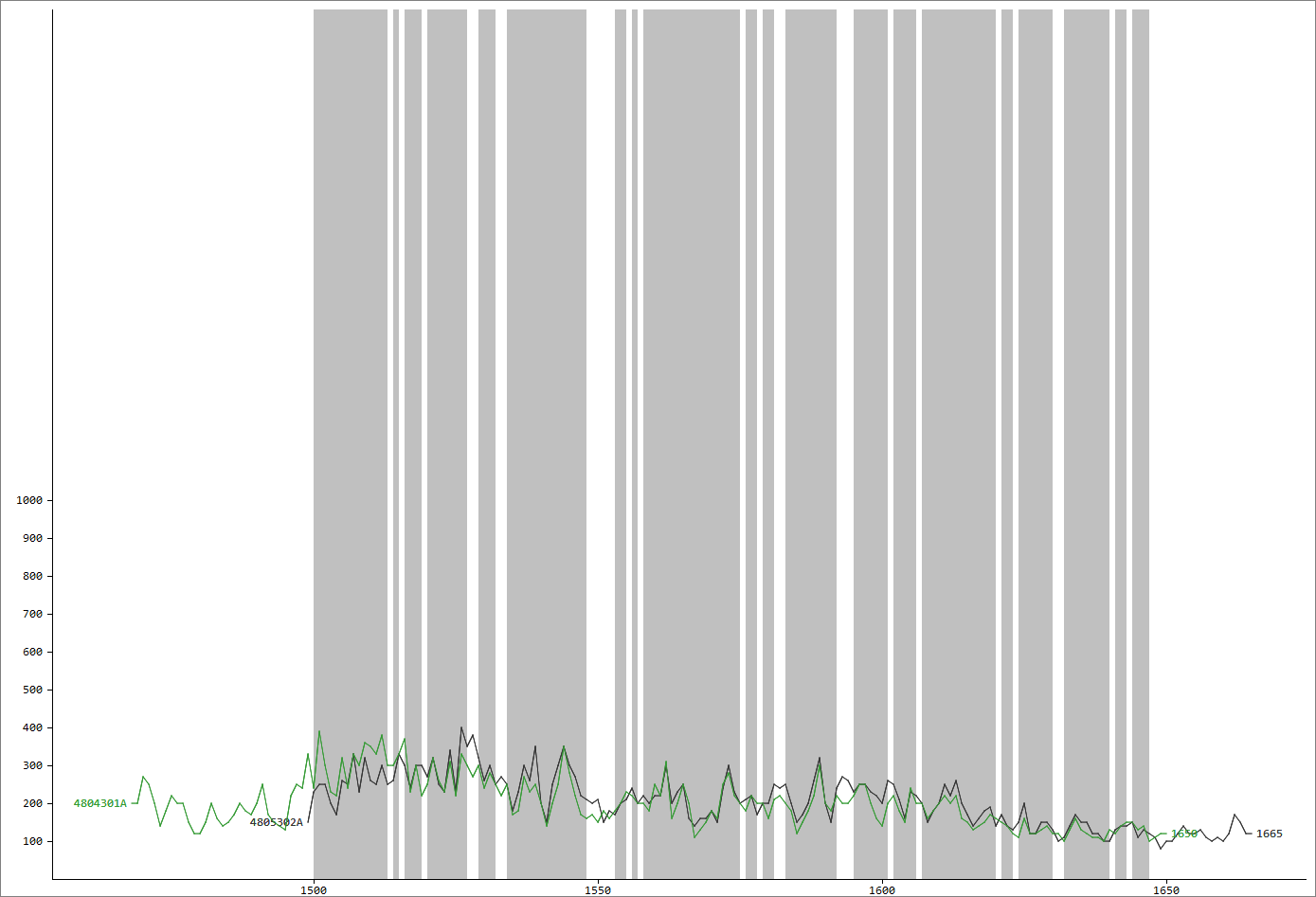


## 12


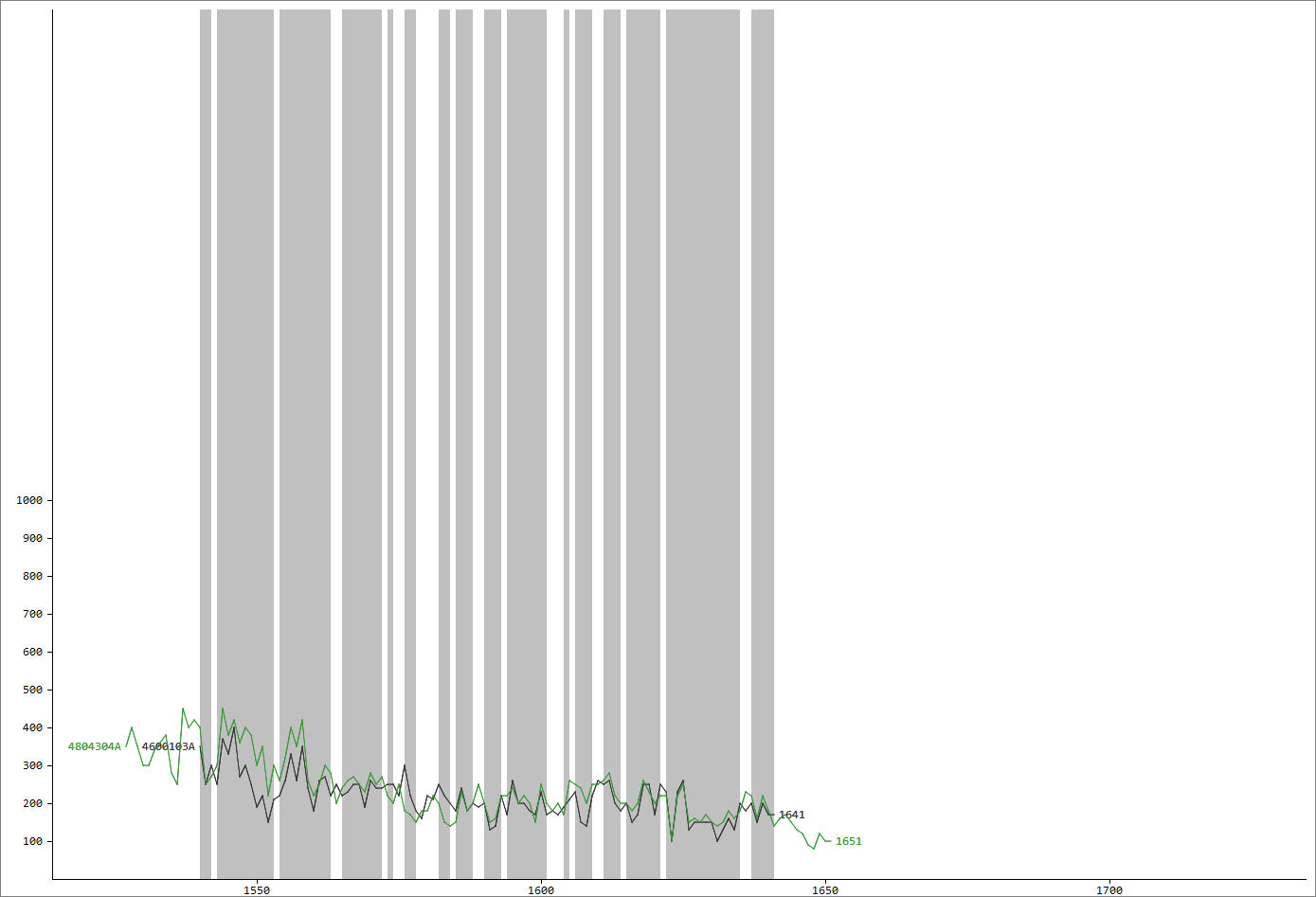


## 13


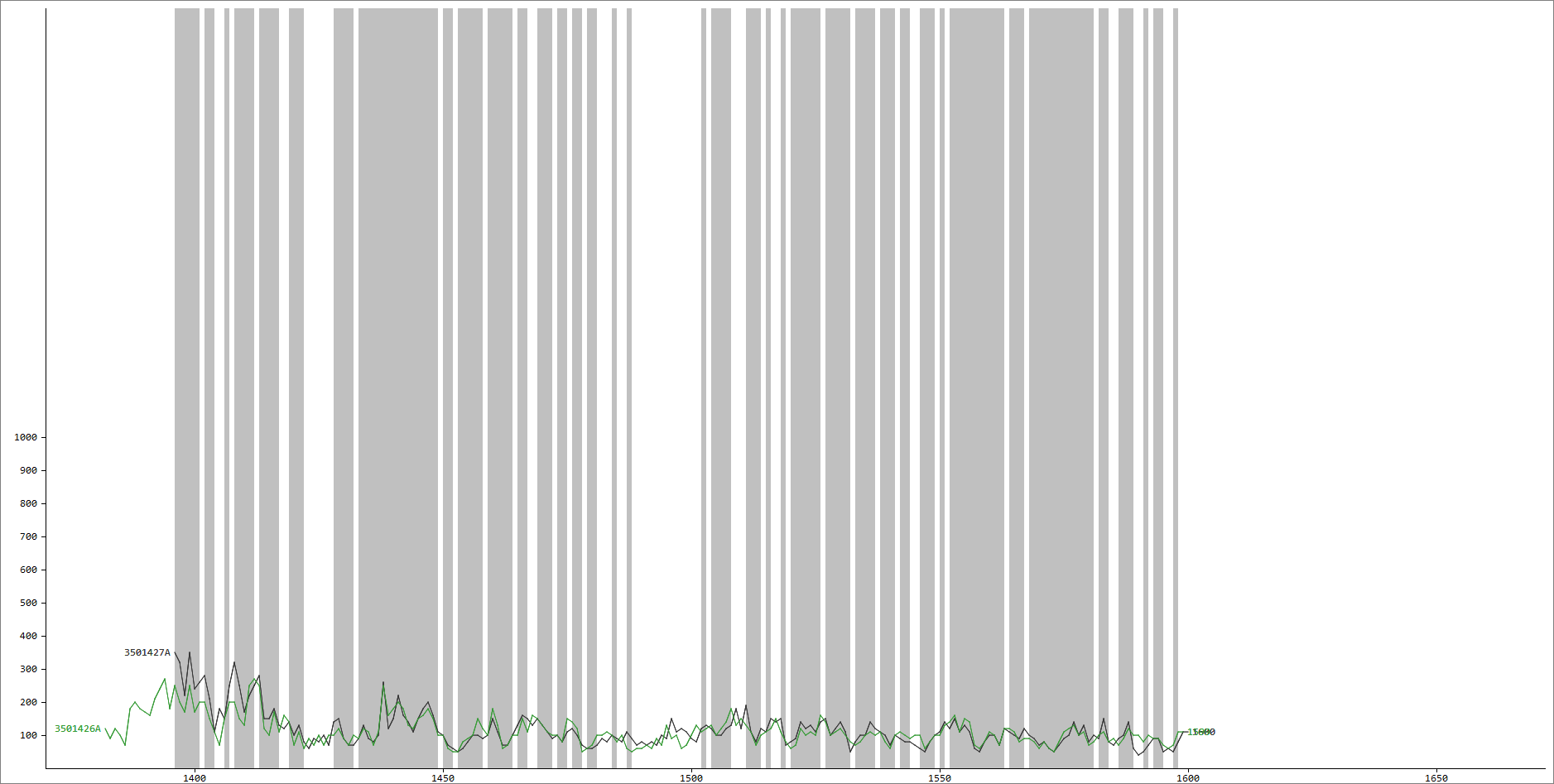


## 14


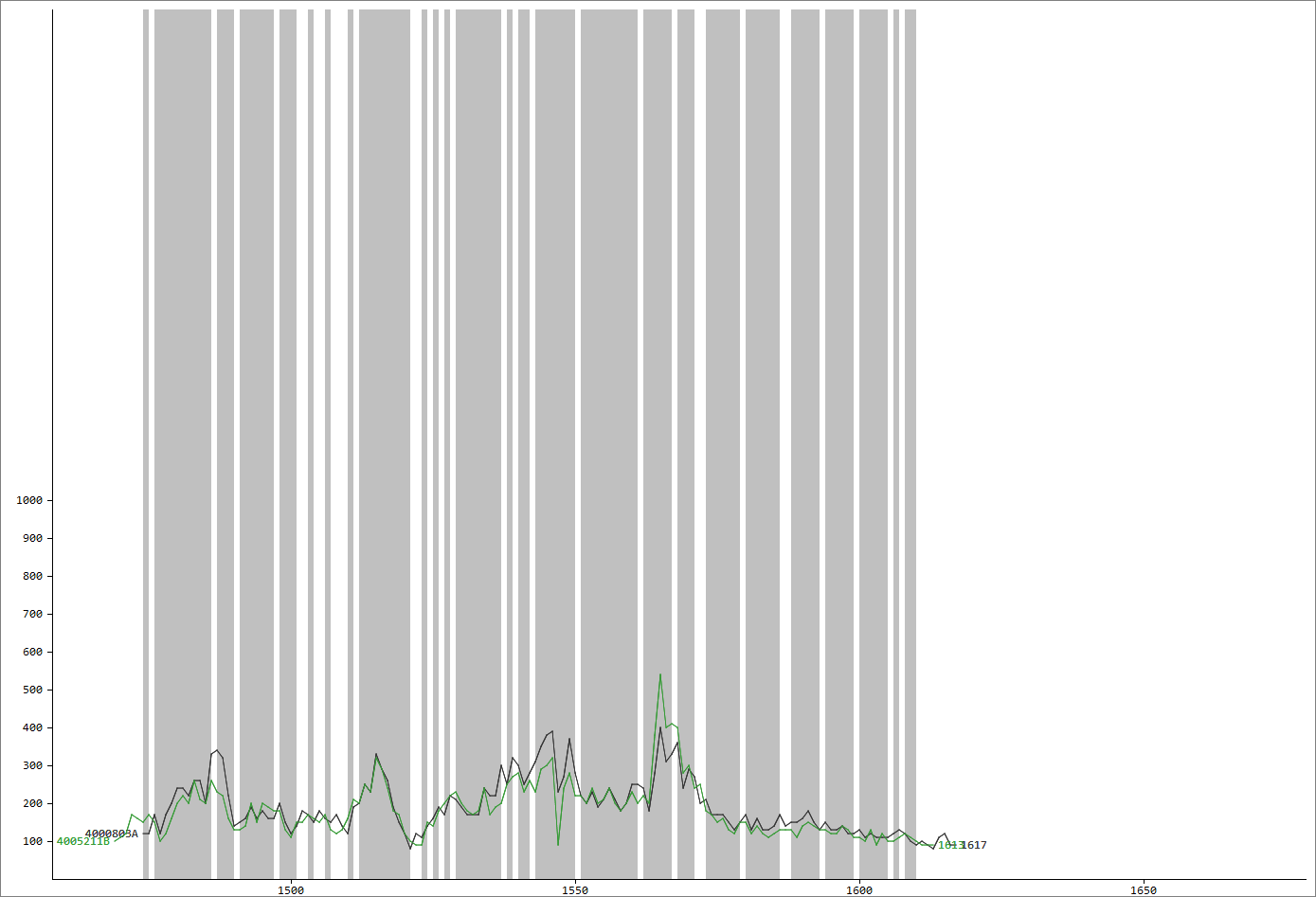


## 15


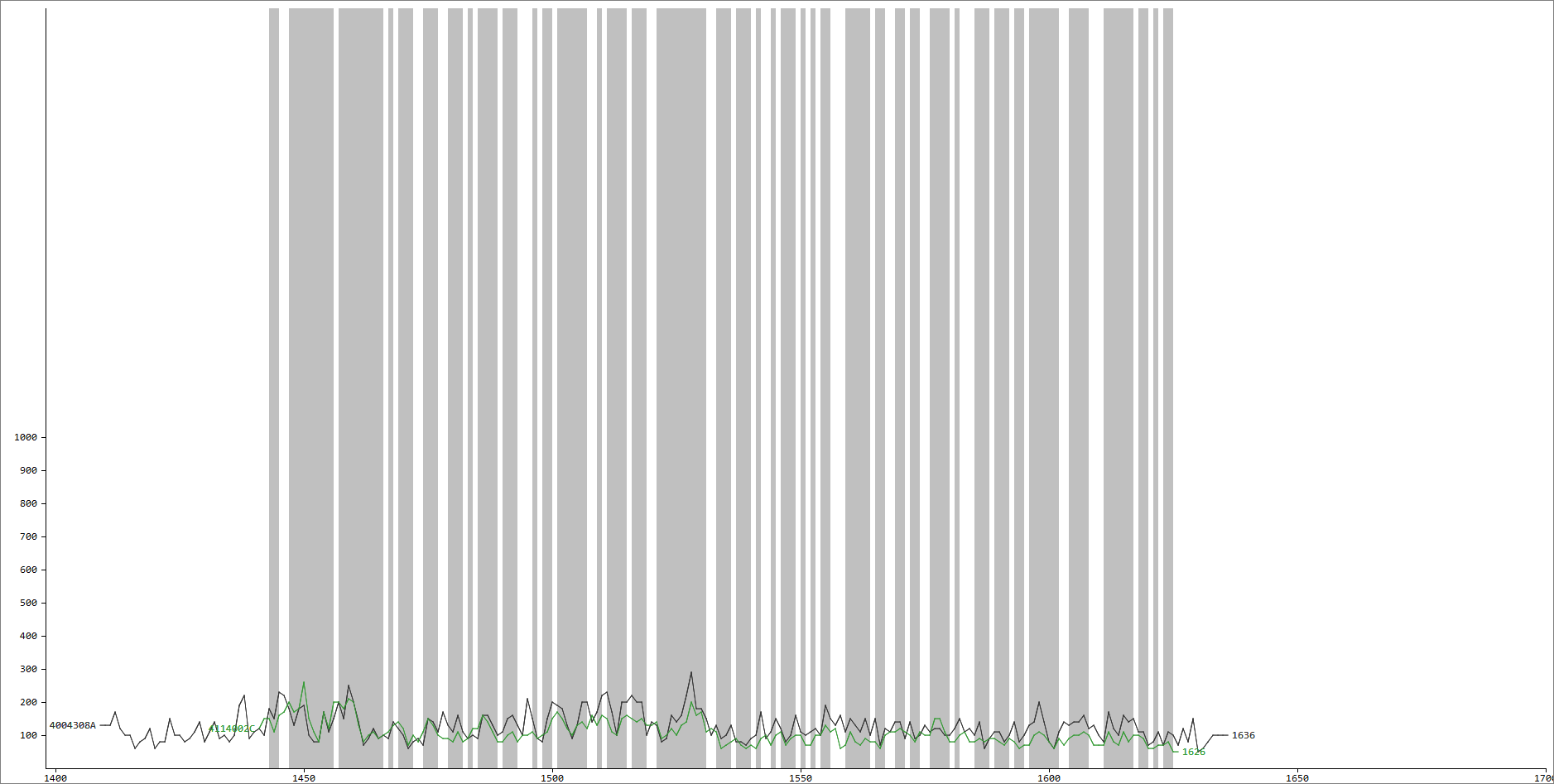


## 16


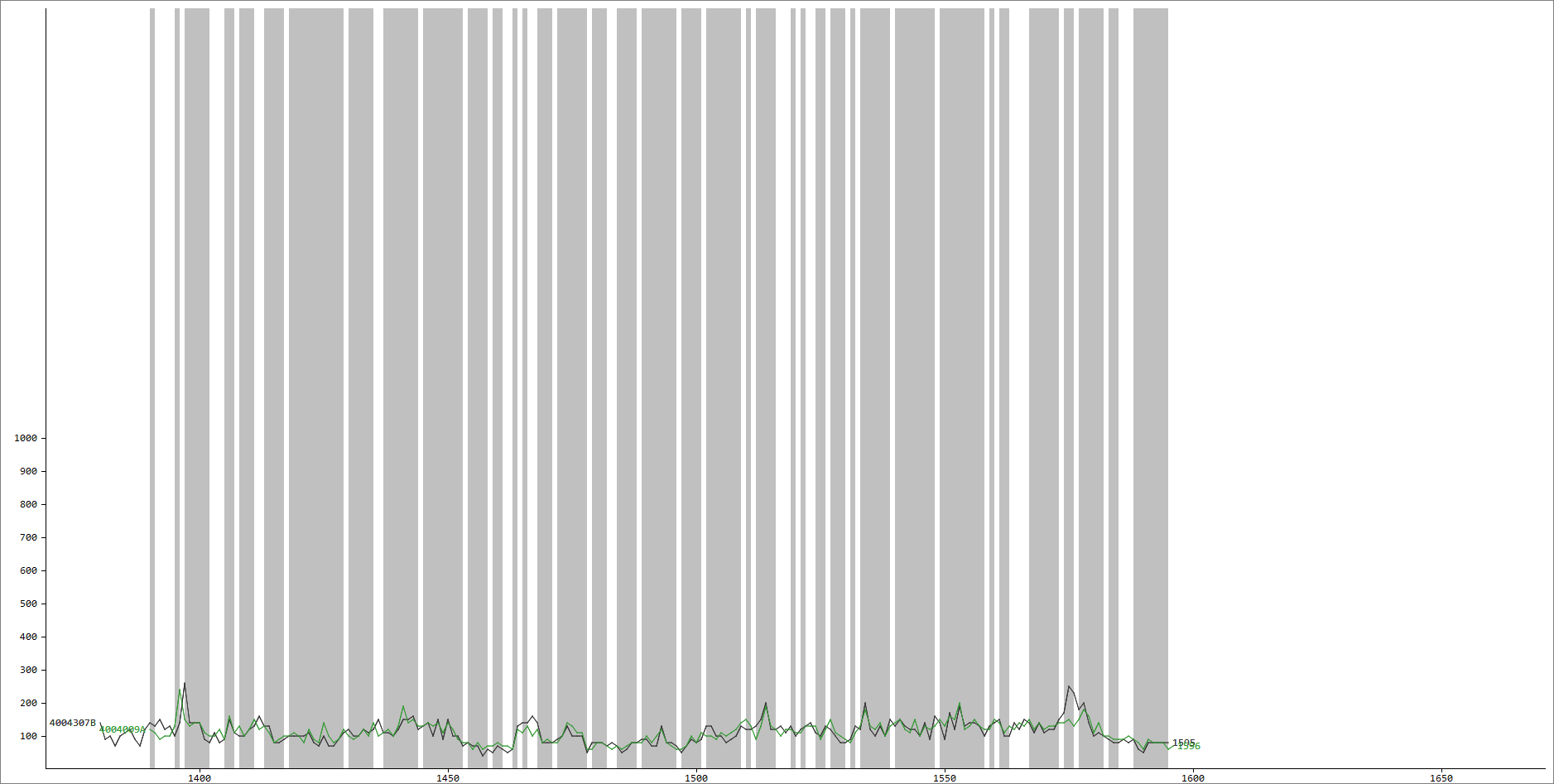


## 17


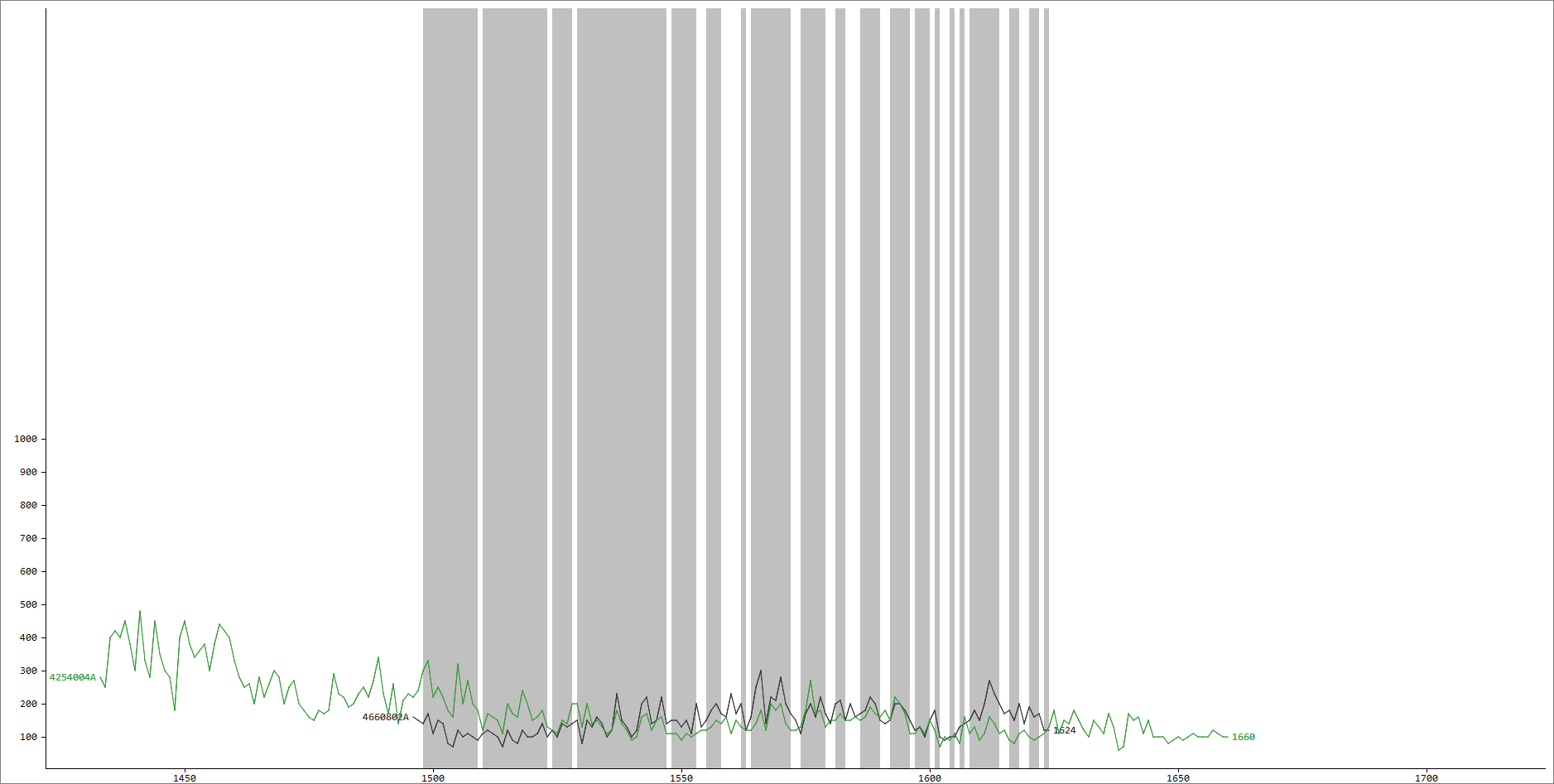


## 18


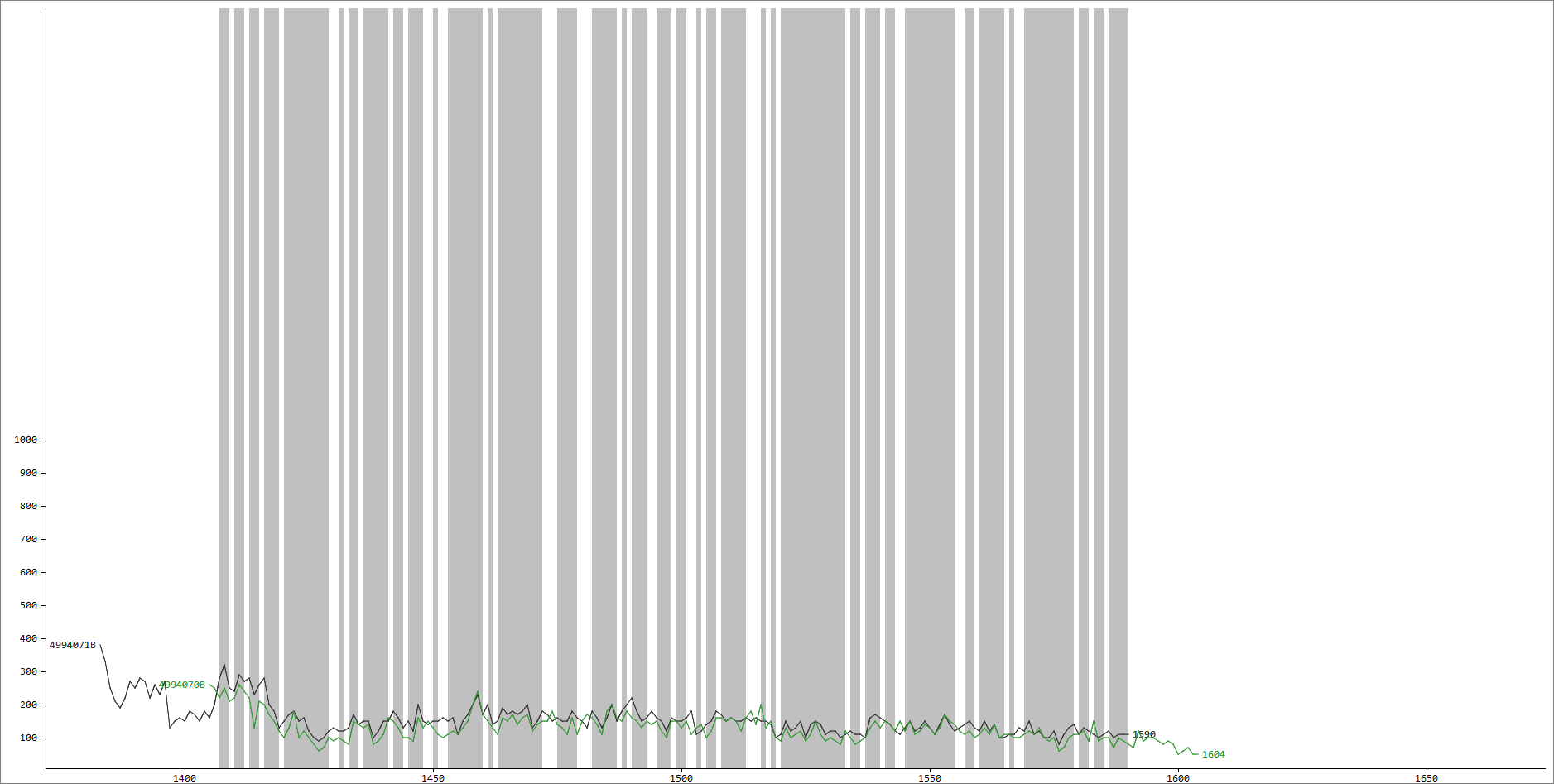


## 19


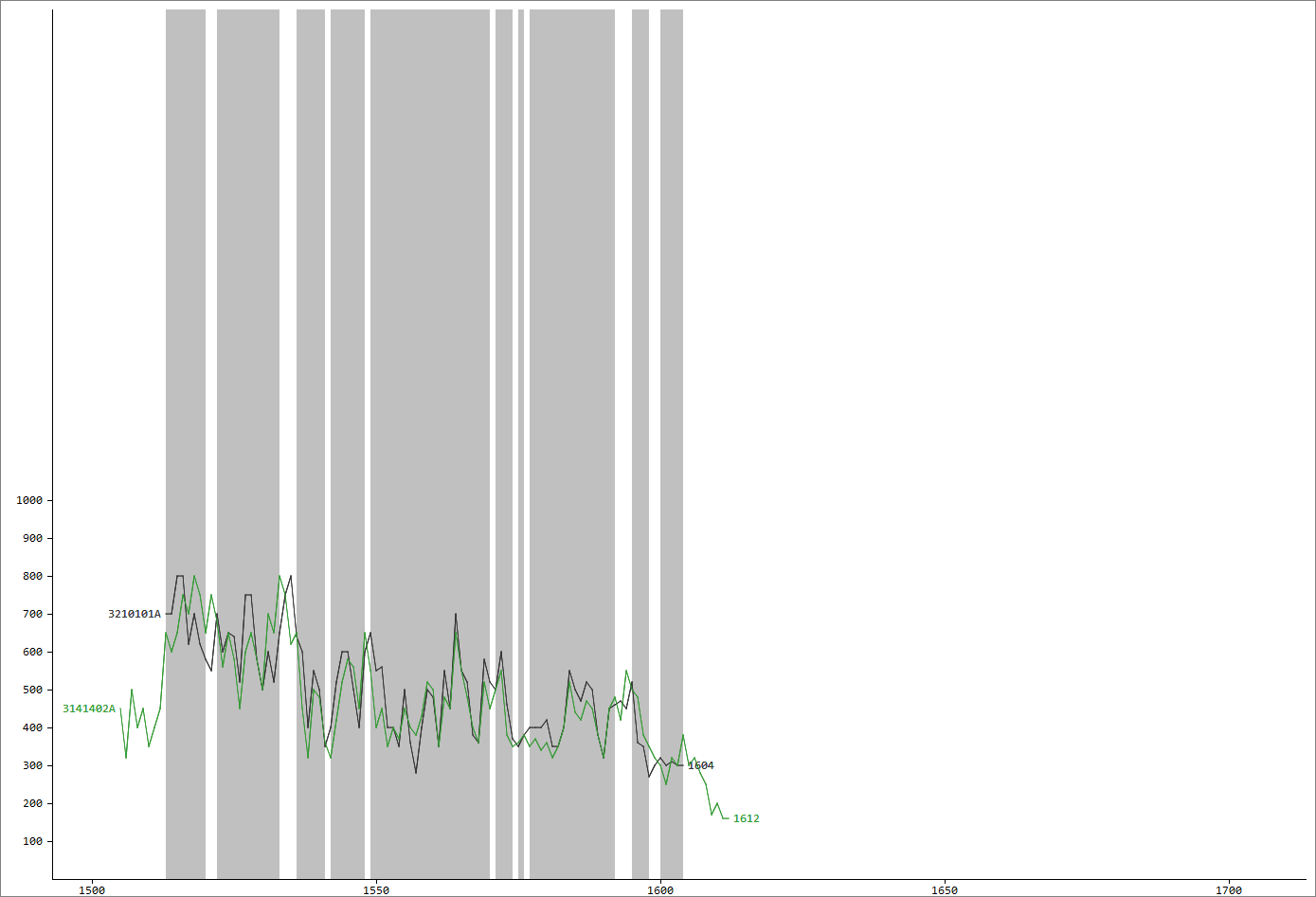


# Groups^[[2]](#footnote-2)^

## GROUP01


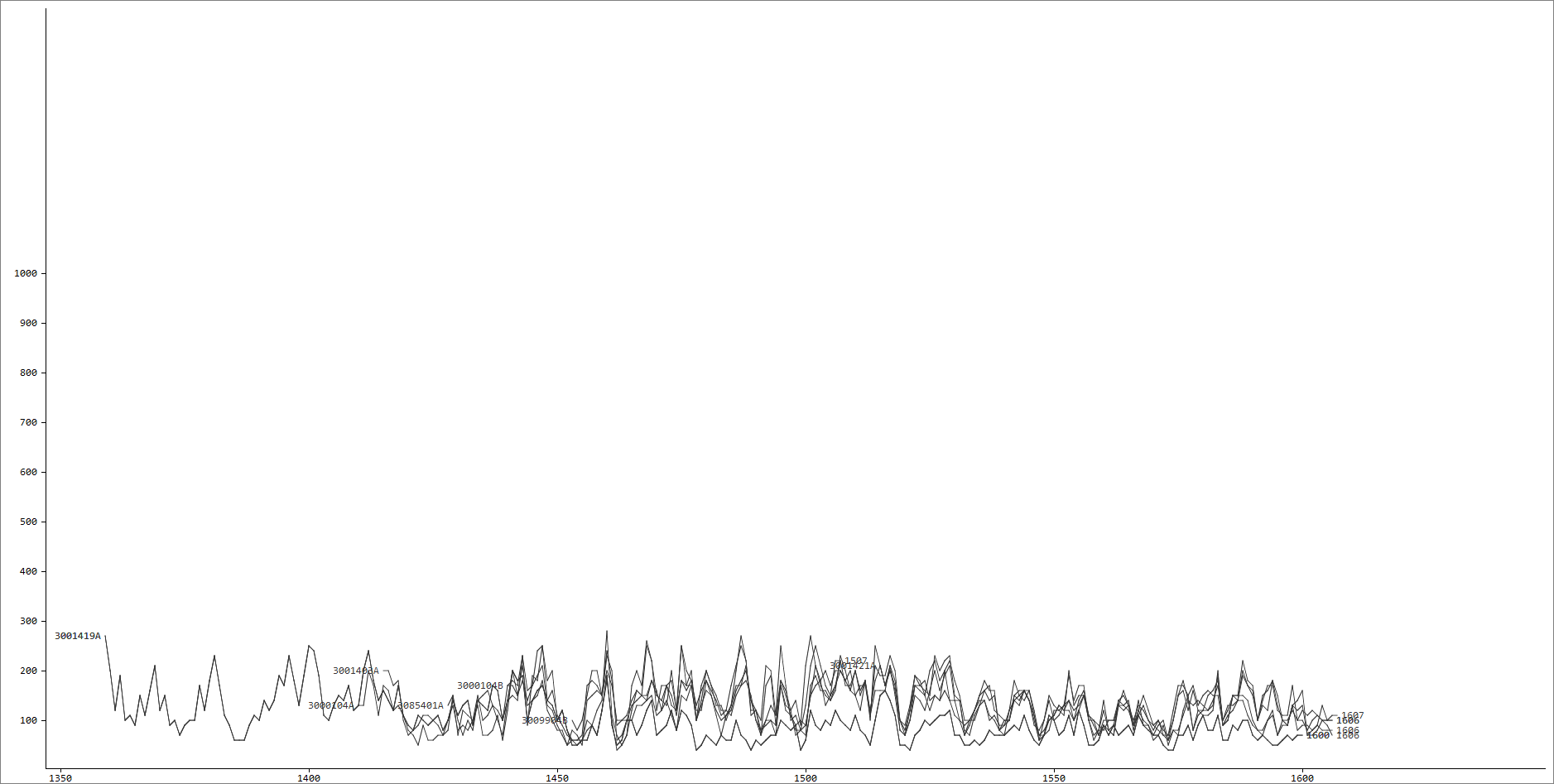


## GROUP02


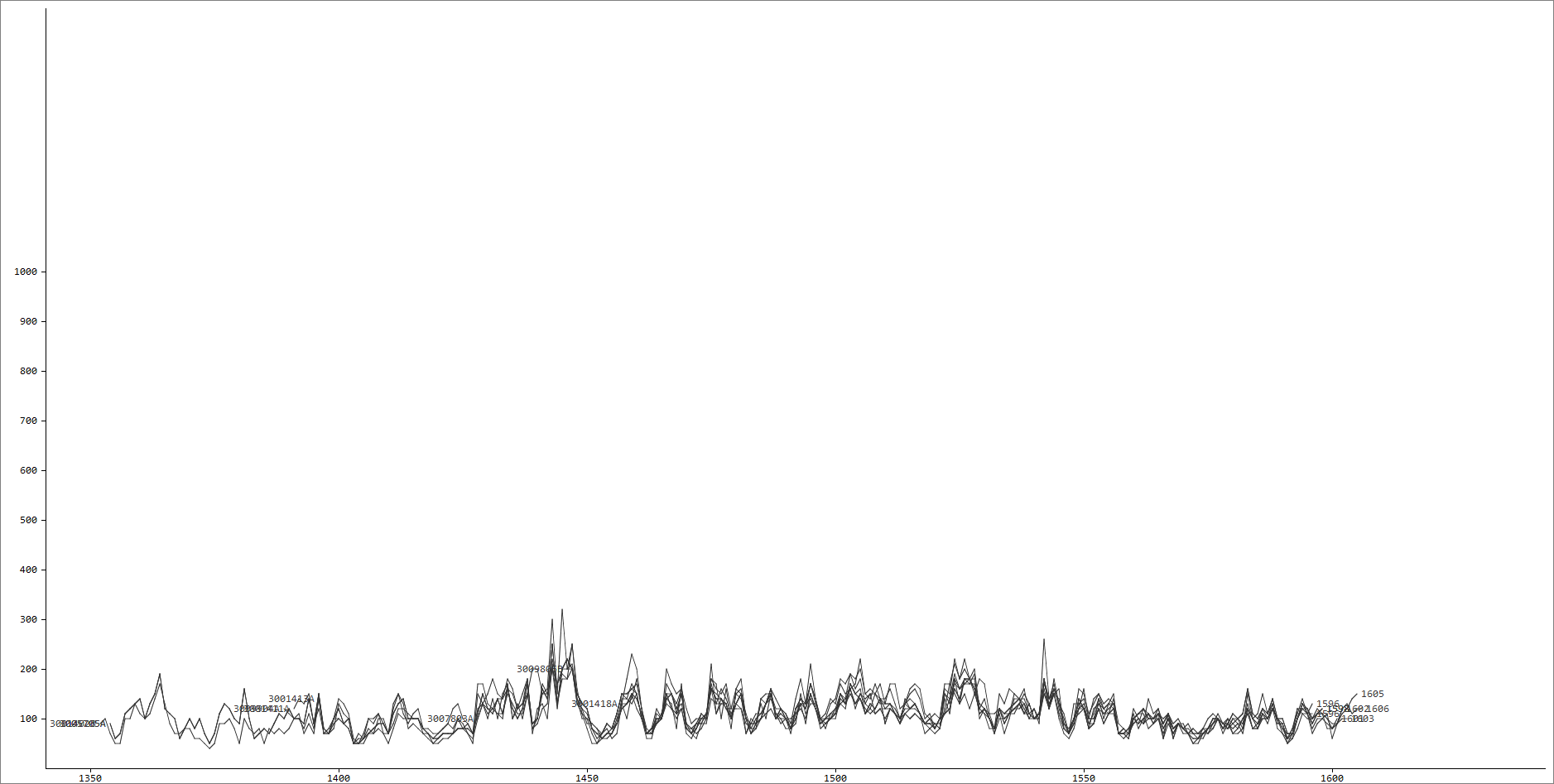


## GROUP03


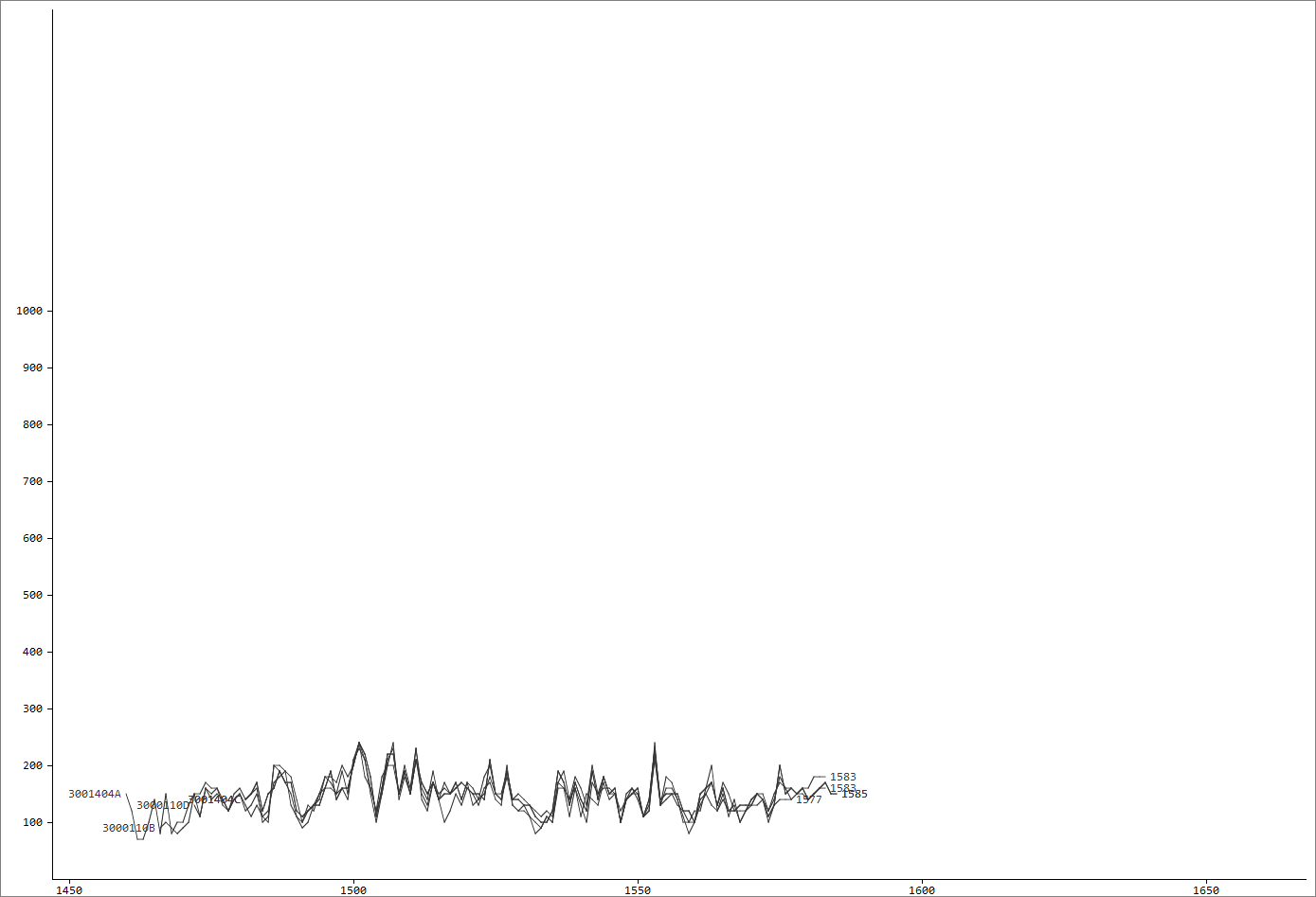


## GROUP04


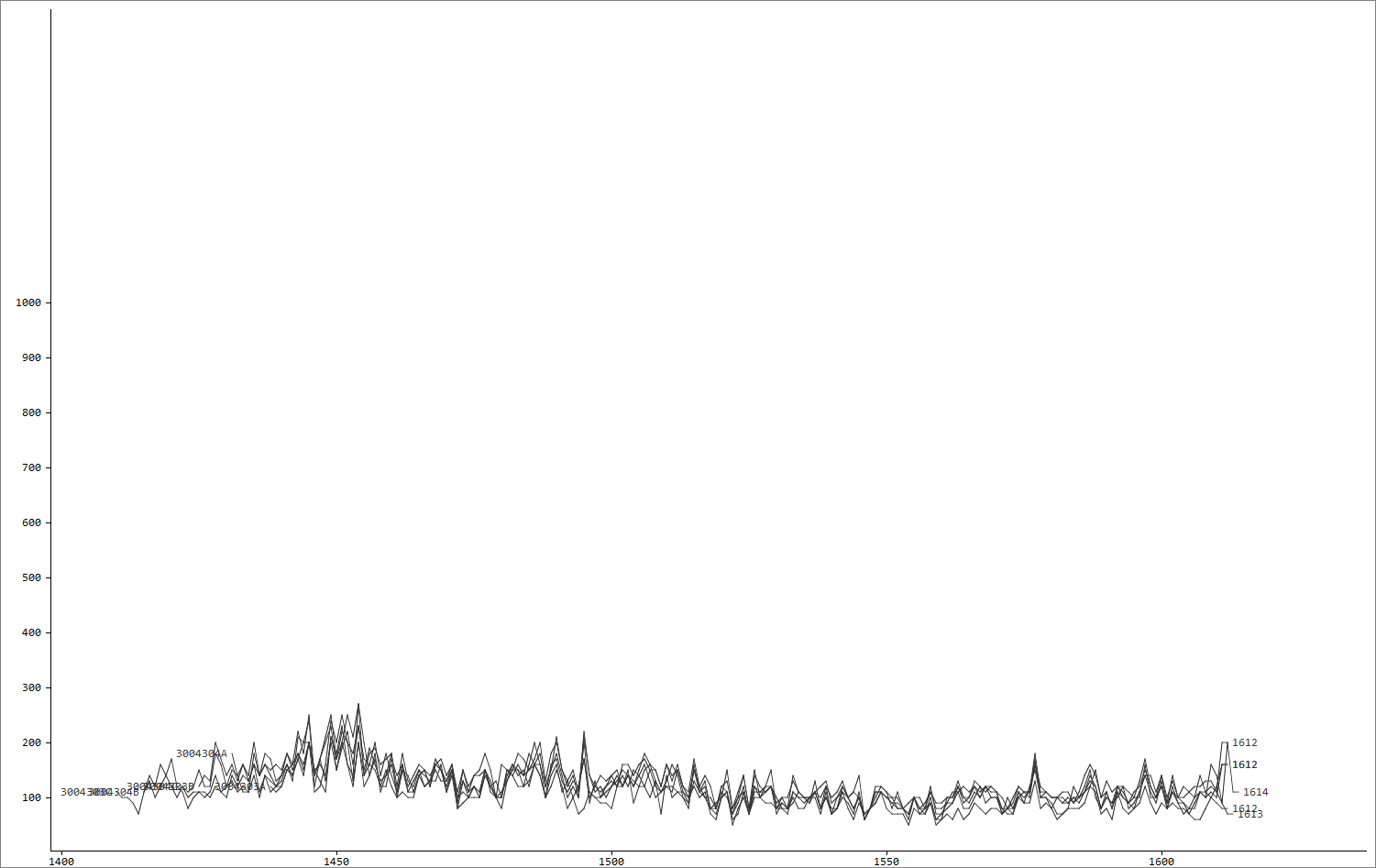


## GROUP05


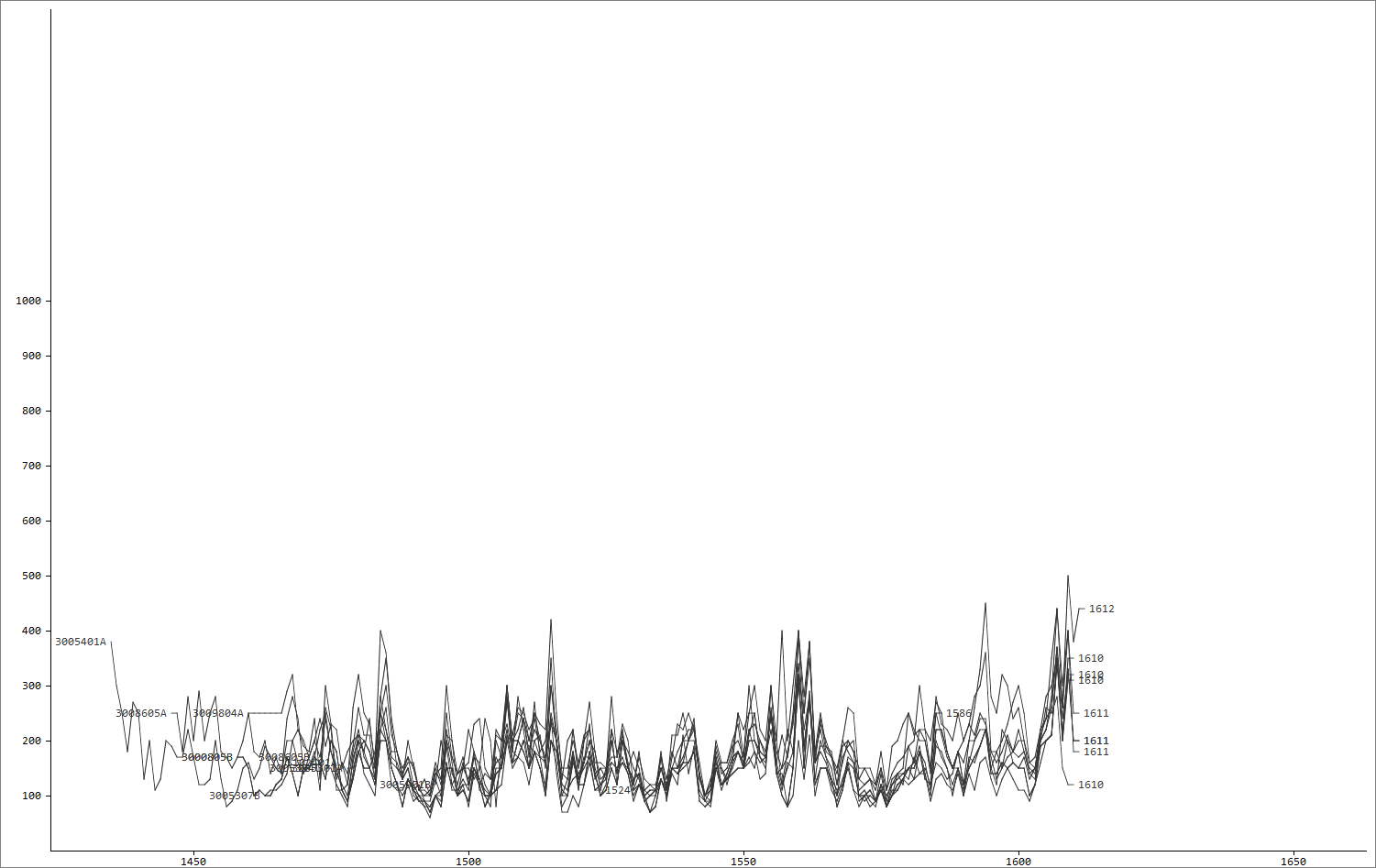


## GROUP06


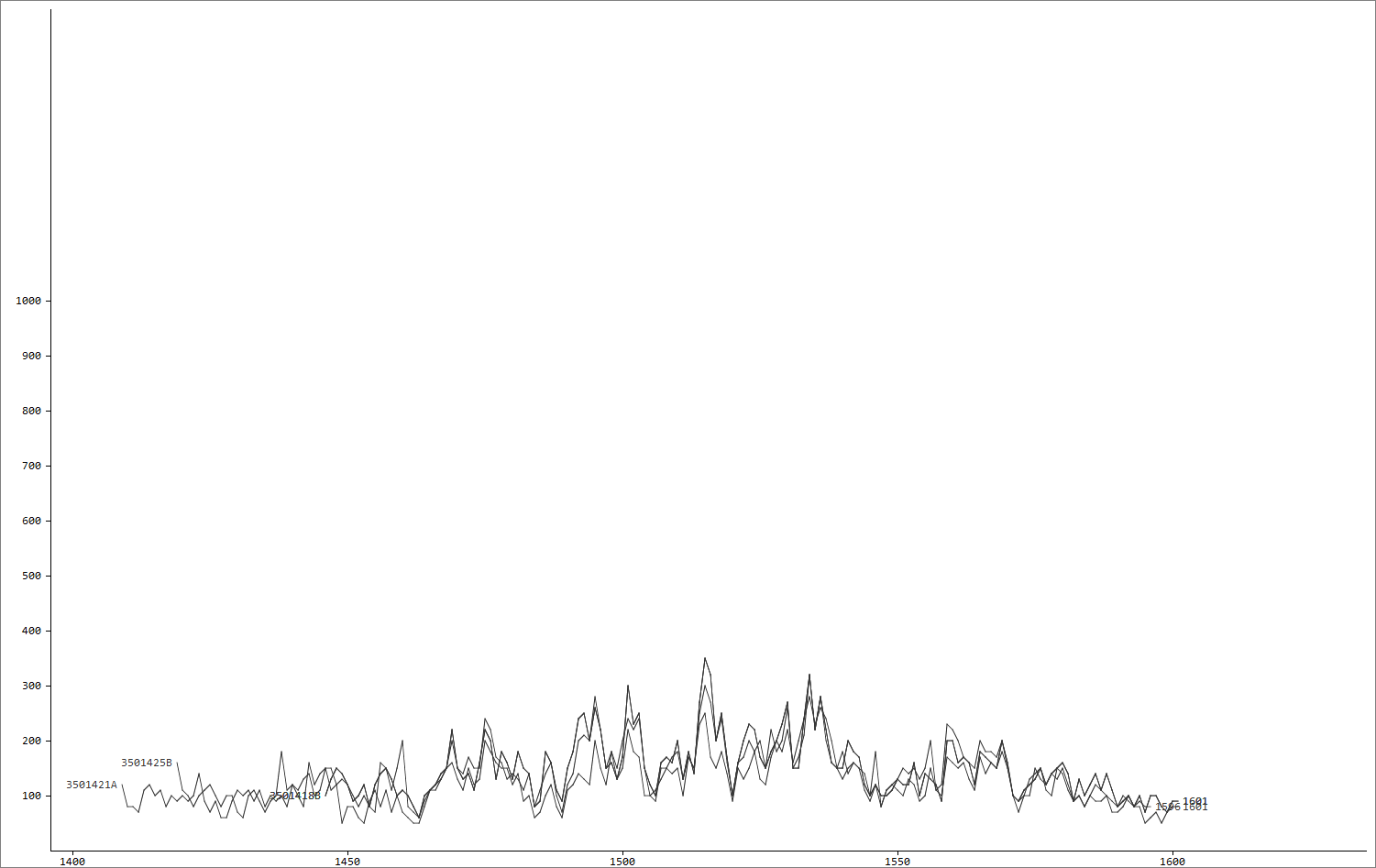


## GROUP07


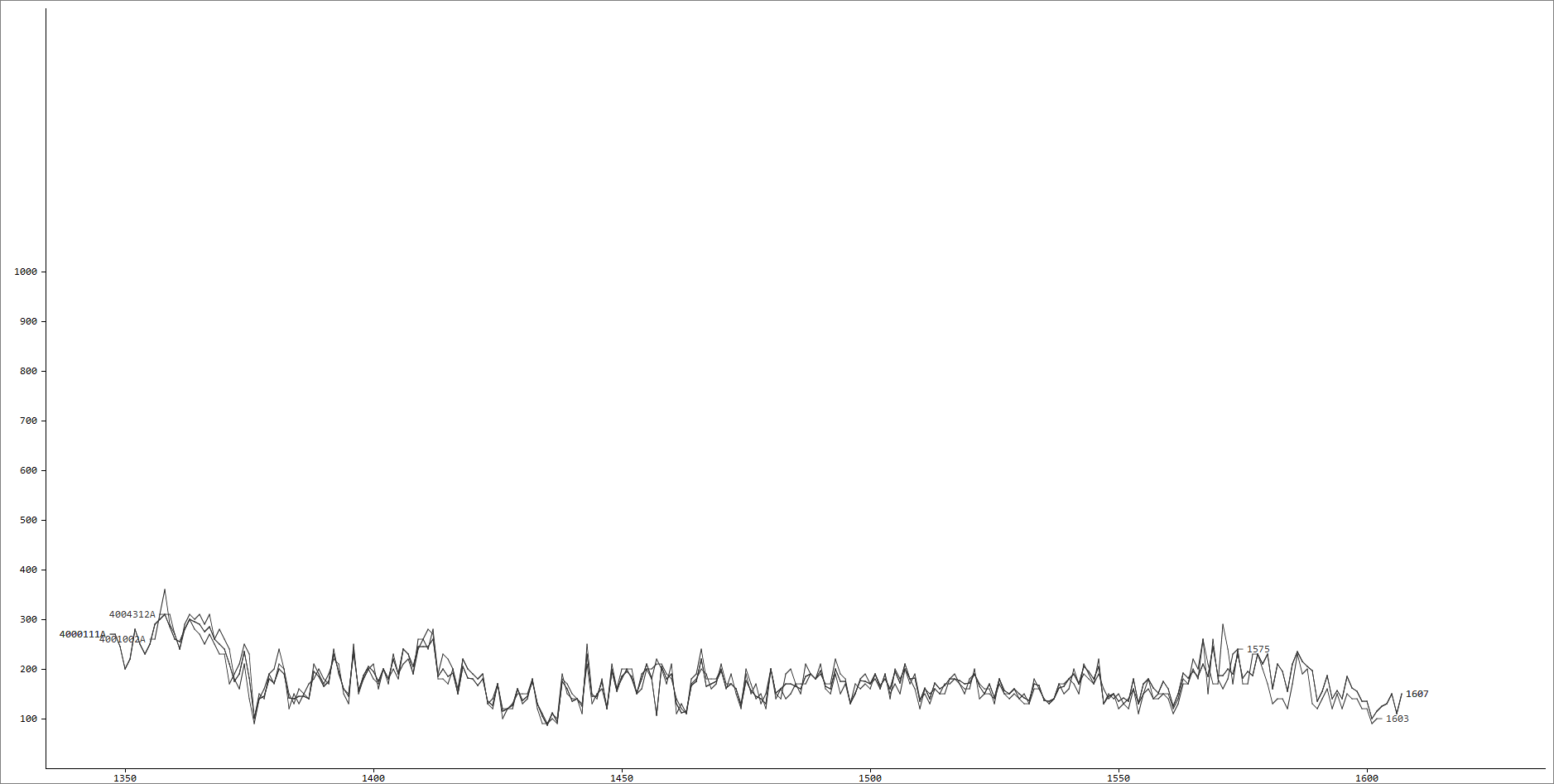


## GROUP08


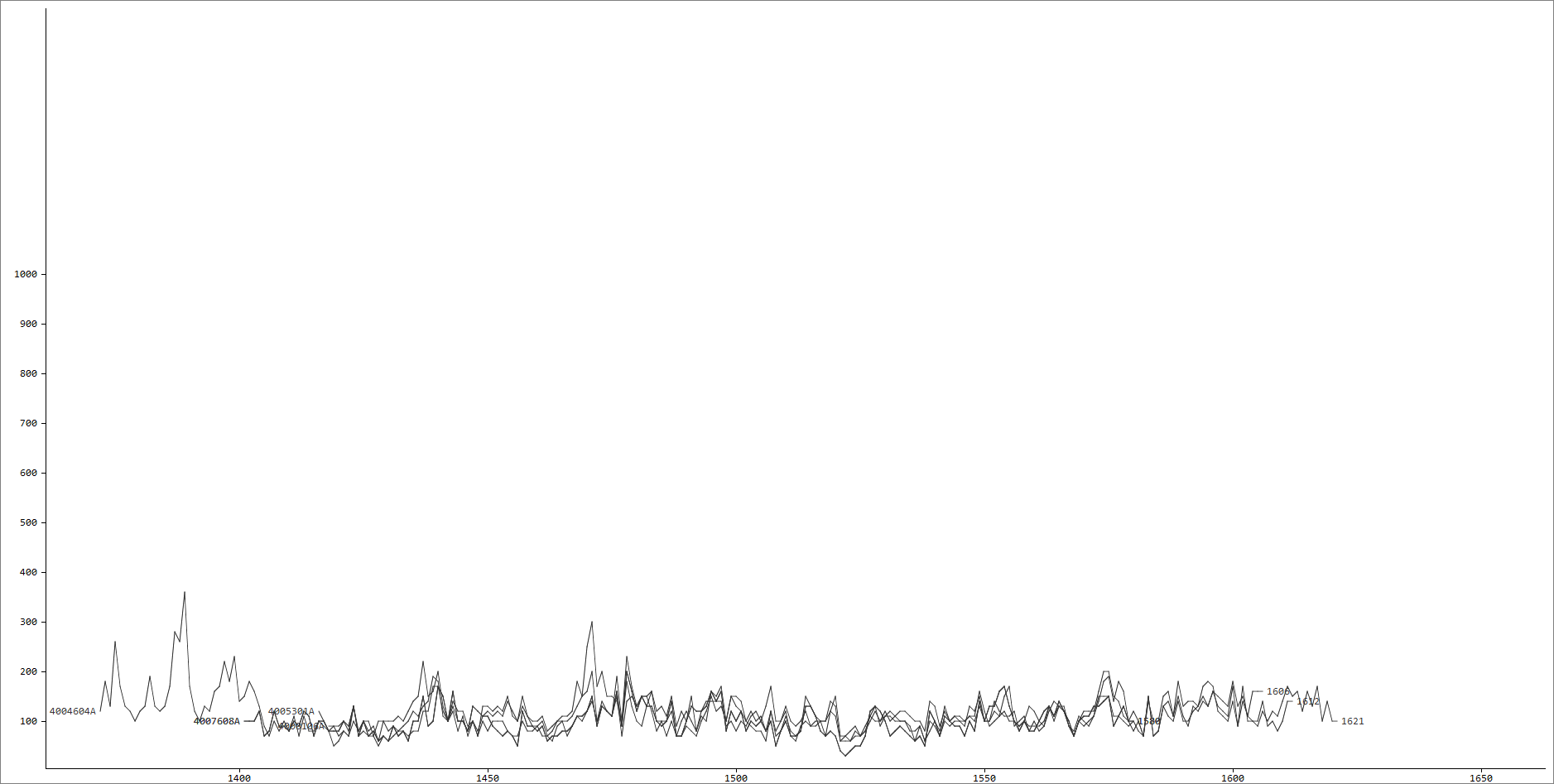


## GROUP09
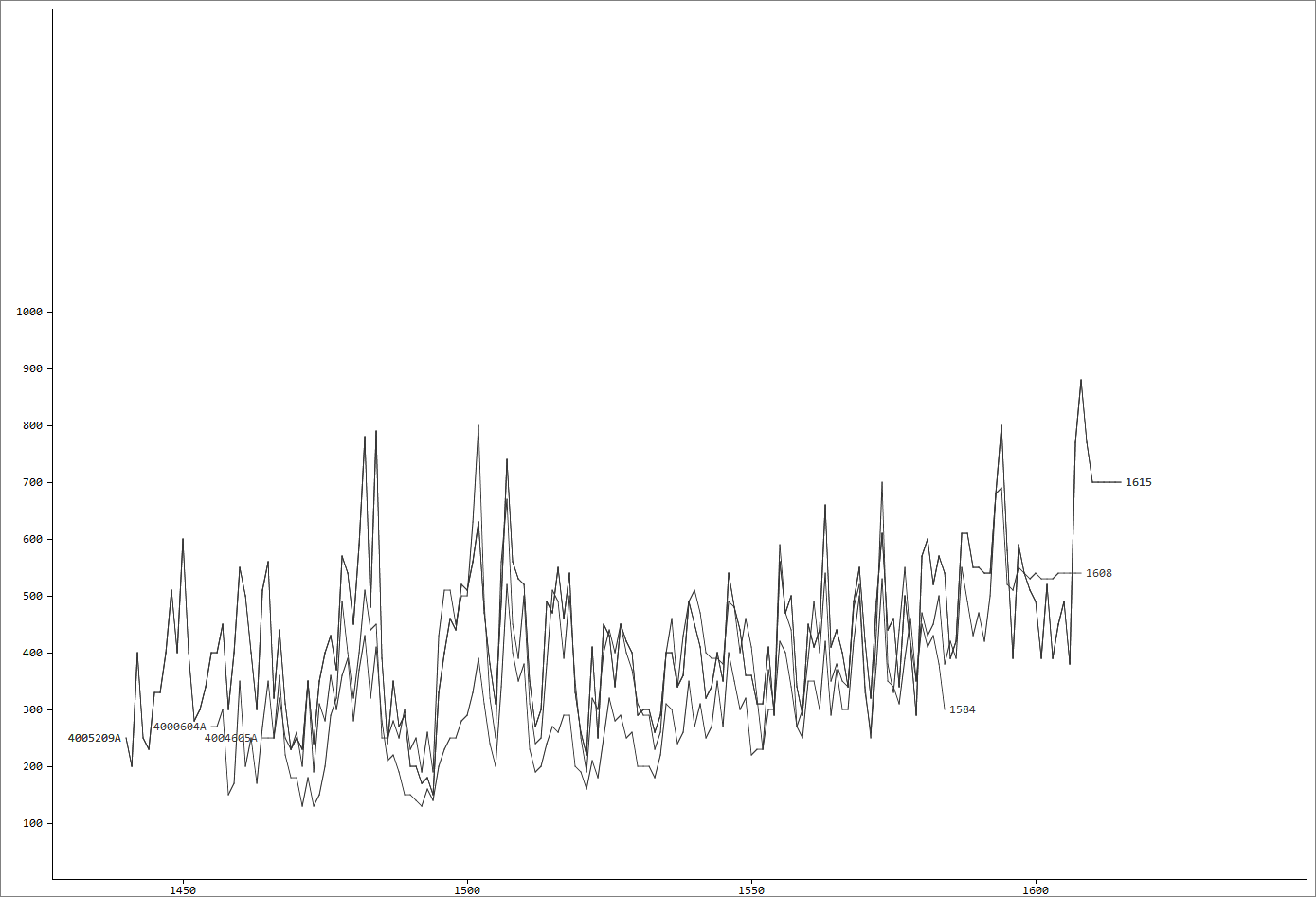


## GROUP10


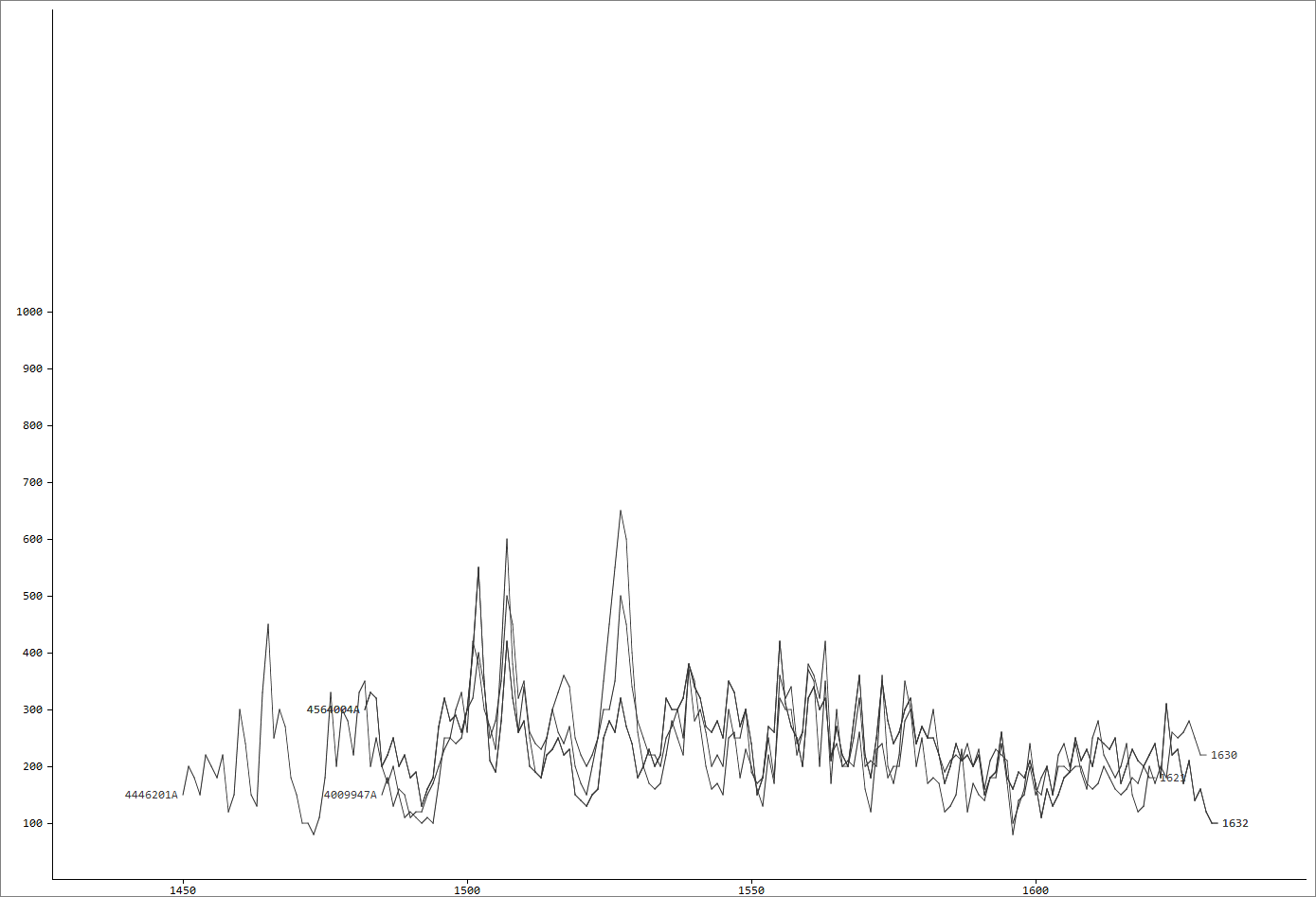


## GROUP11


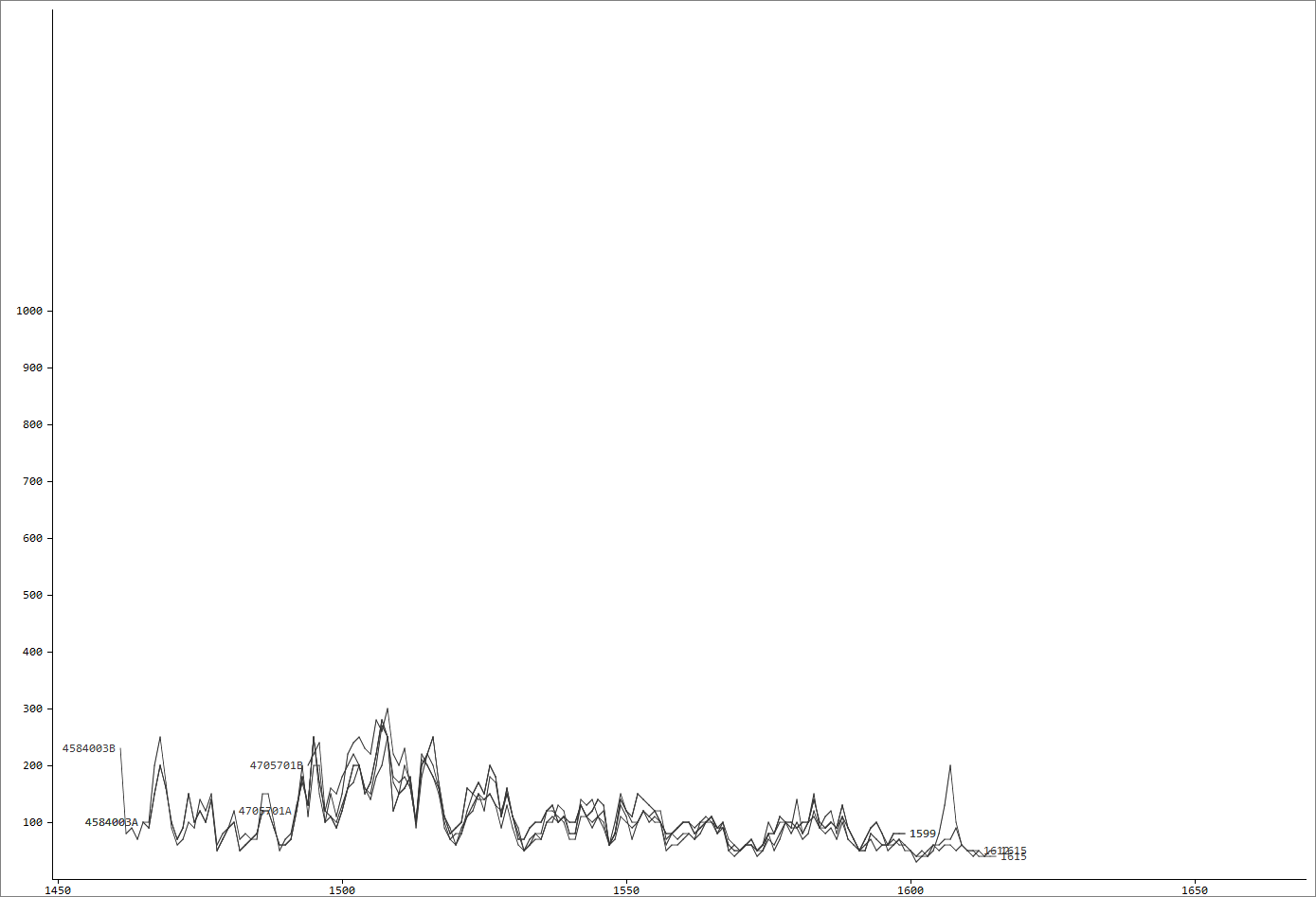


## GROUP12


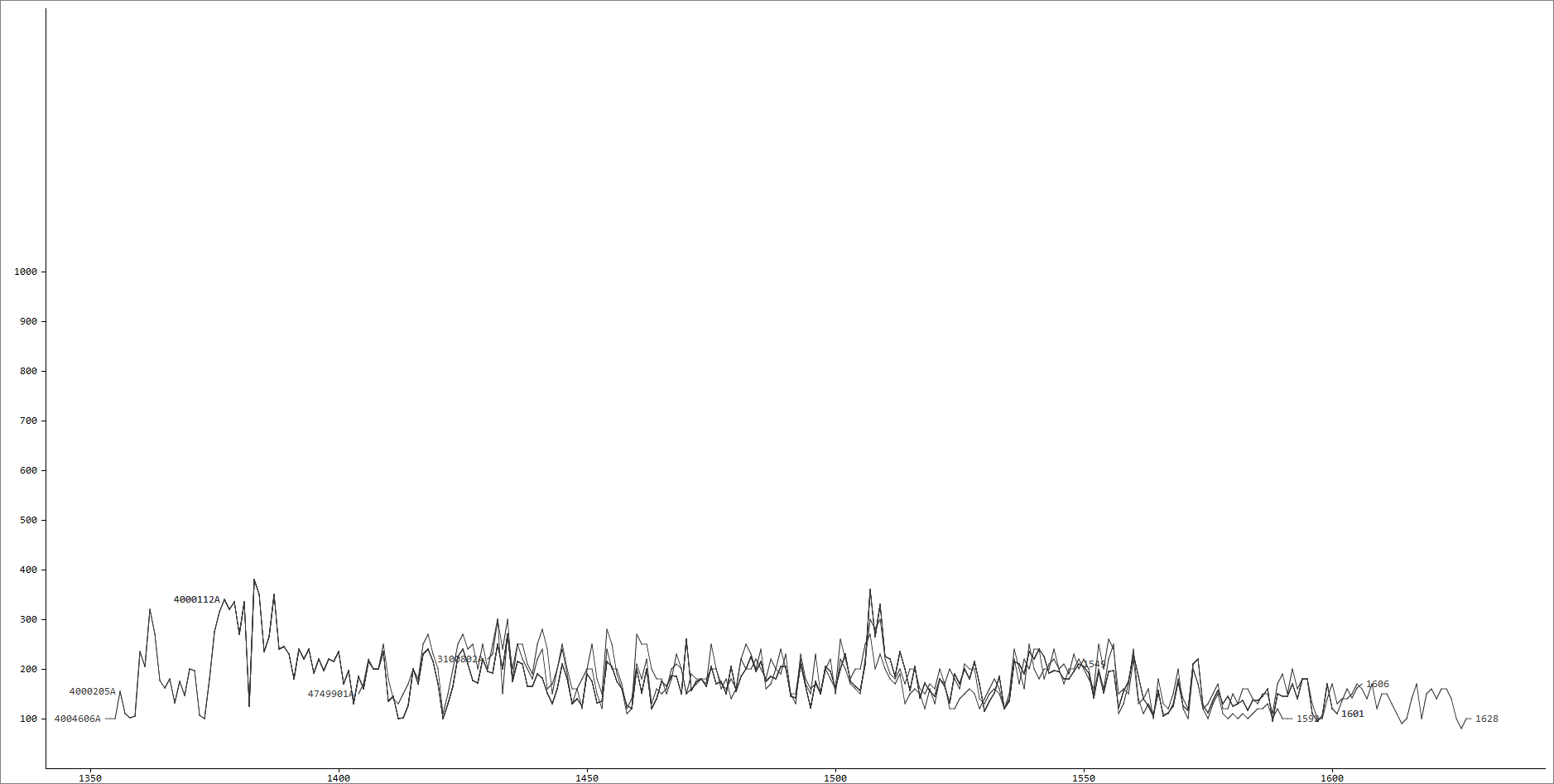


## GROUP13


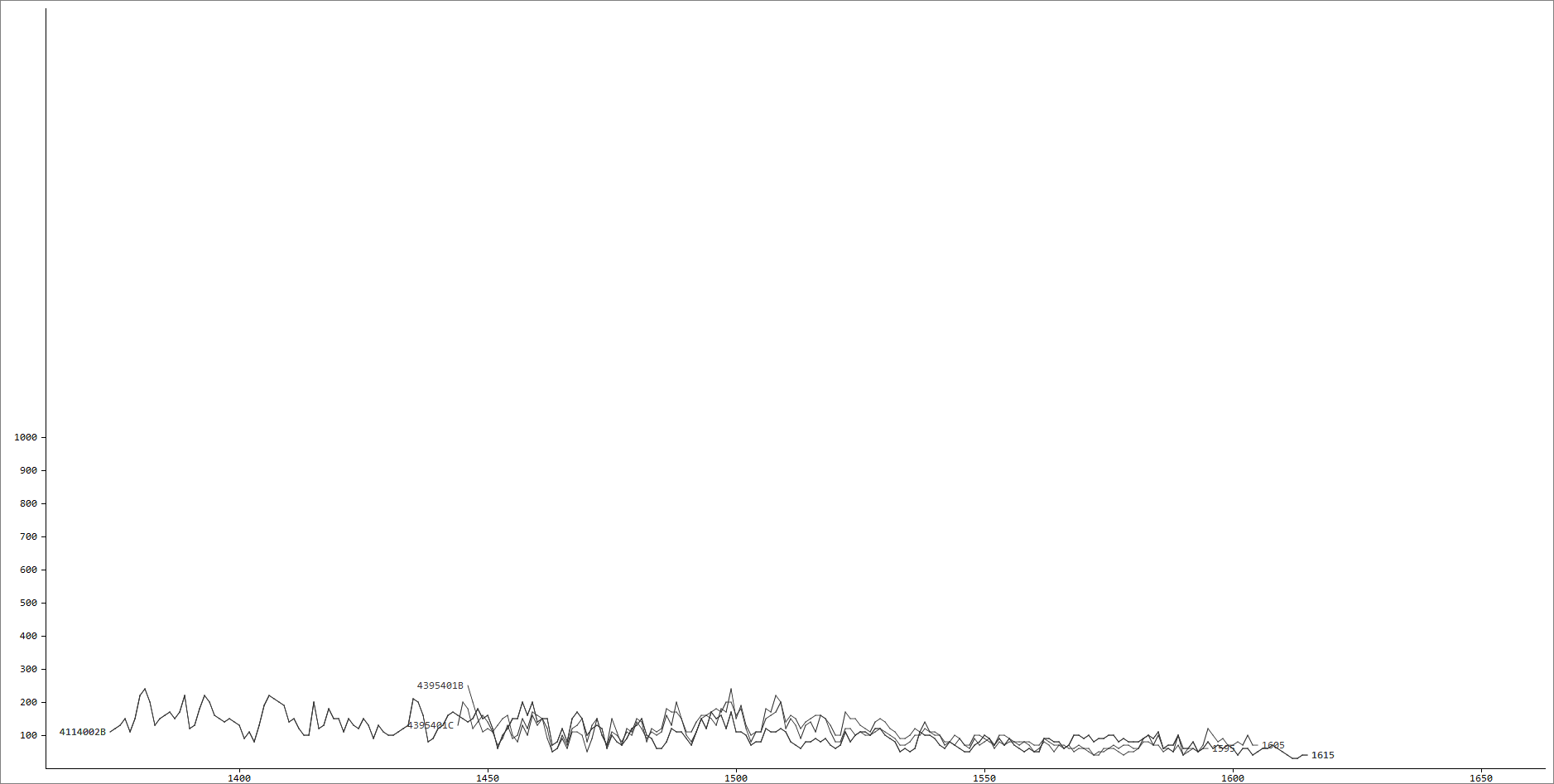


## GROUP14


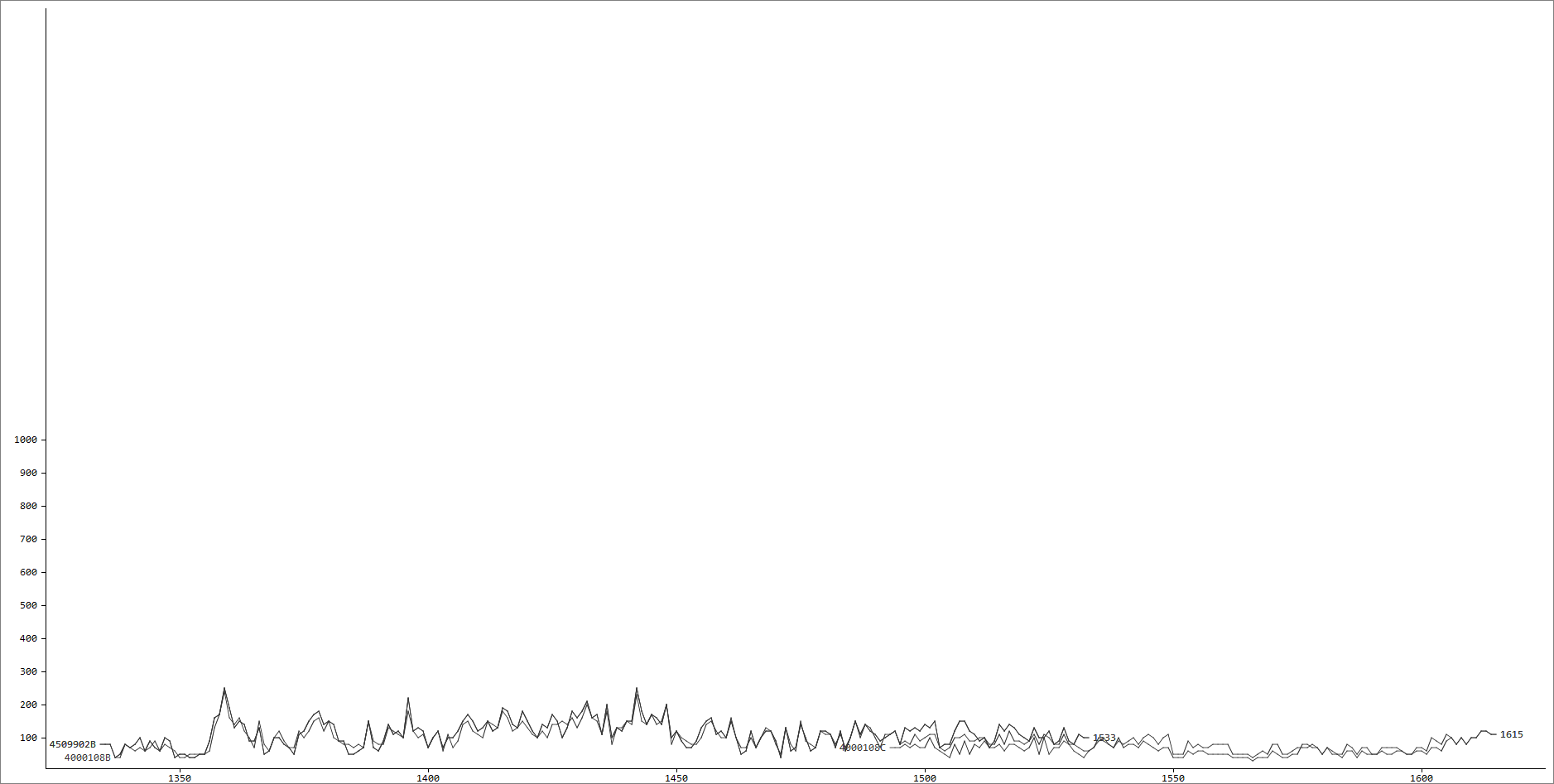


## GROUP15


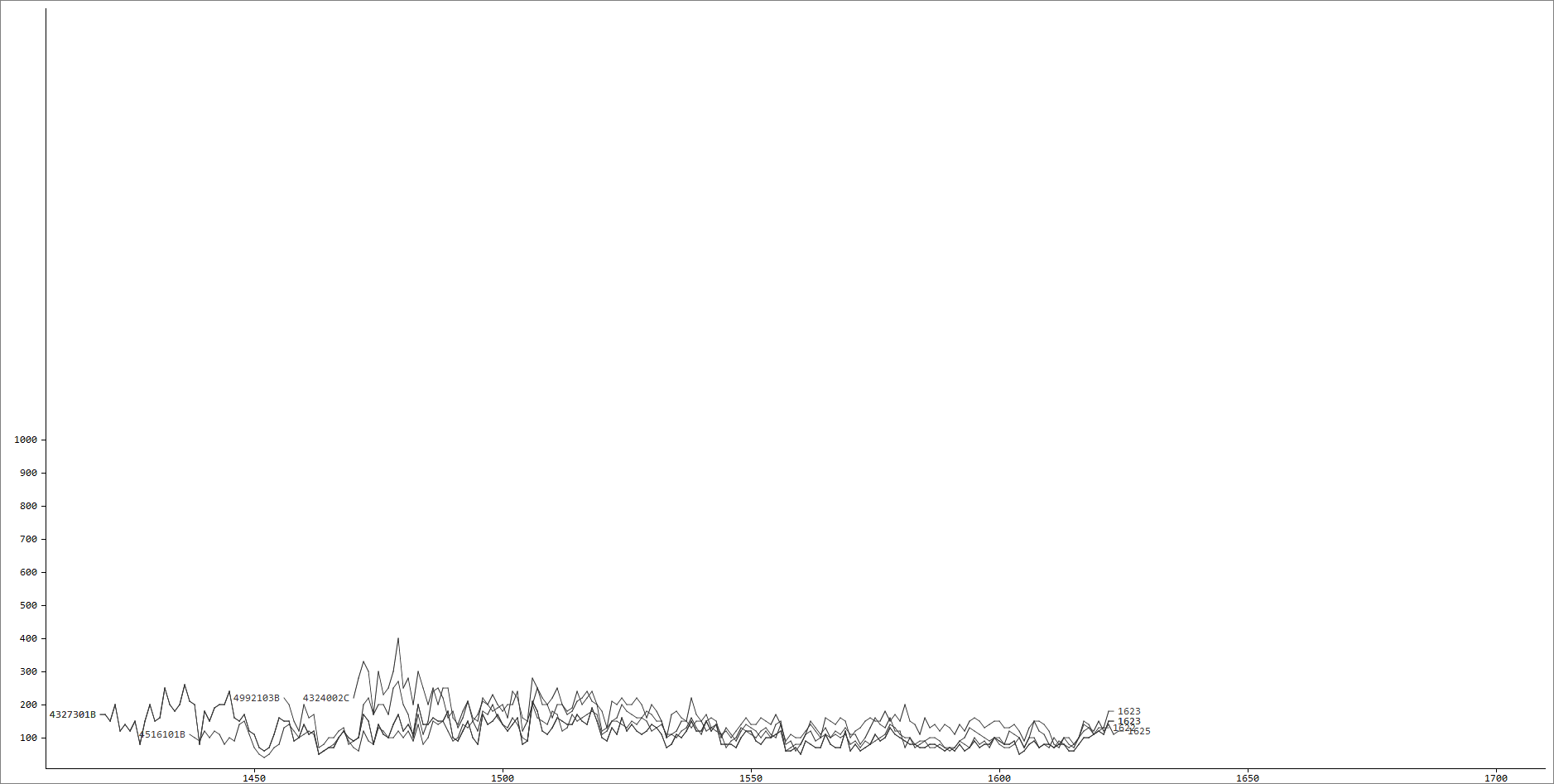


1. Graphs of the pairs of boards showing the visual match between the tree-ring series of each board. The shaded area shows the percentage of parallel variation (%PV) between the tree-ring series. Y-axis: ring-width (1/100 mm); x-axis: calendar years. [↑](#footnote-ref-1)
2. Graphs of the pairs of boards showing the visual match between the tree-ring series of each board. Y-axis: ring-width (1/100 mm); x-axis: calendar years. [↑](#footnote-ref-2)
